# Host immunosenescence compromises *Mycobacterium tuberculosis* clearance

**DOI:** 10.18632/aging.206374

**Published:** 2026-05-04

**Authors:** Falak Pahwa, Shweta Chaudhary, Ashish Gupta, Shivam Chaturvedi, Ranjan Kumar Nanda

**Affiliations:** 1 Translational Health Group, International Centre for Genetic Engineering and Biotechnology, New Delhi, 110067, India; 2 Bio-experimentation Facility, International Centre for Genetic Engineering and Biotechnology, New Delhi, 110067, India

**Keywords:** aging, tuberculosis, immunosenescence, T_FH_ cells, proteomics

## Abstract

Immunosenescence increases susceptibility to infectious diseases like tuberculosis (TB) in older adults (≥60 years) and hinder effective containment of *Mycobacterium tuberculosis* (Mtb) during therapeutic intervention. A comprehensive understanding of the cellular and molecular changes underlying age-associated immune alterations may inform development of strategies to improve treatment outcomes. Here, we monitored the immunopathology, frequency, and functionality of immune cells across extreme age groups of C57BL/6 mice following low aerosol dose infection (100-120 cfu) with Mtb H37Rv and treatment with rifampicin and isoniazid (RIF-INH). Up to 6 weeks post infection, mycobacterial load in tissues (lung, spleen, and liver) of old (17-19 months; M) and aged (31M) C57BL/6 mice was similar to that of young (2-4M) mice. However, at two weeks post-treatment, older mice showed a slower rate of Mtb clearance in the lungs. Mtb-infected old mice had higher splenic T-follicular cytotoxic (T_FC_)-like cells, and proteomic analysis of flow-sorted CD4^+^CD44^+^ T cells revealed deregulated mitochondrial proteins (4-hydroxy-2-oxoglutarate aldolase, aspartate aminotransferase, and prostaglandin E synthase), suggesting impaired mitochondrial function. Collectively, these findings suggest that age-associated immune alterations may disrupt immunometabolic pathways, thereby contributing to the delayed Mtb clearance. Targeting immunometabolic dysfunction therefore represents a promising strategy to enhance TB treatment efficacy and reduce disease burden in older populations.

## INTRODUCTION

A recent WHO report predicts that, by 2030, one in six people globally will be over 60 years of age [1]. Aging profoundly impairs immune competence, a phenomenon termed immunosenescence, rendering older adults (≥60 years) highly vulnerable to infectious diseases such as tuberculosis (TB) [2]. *Mycobacterium tuberculosis* (Mtb), the causative pathogen of TB, induces hypermethylation and transcriptional reprogramming of host immune cells, further exacerbating age-related immune dysfunction [3]. Clinically, older adults exhibit reduced vaccine effectiveness alongside heightened susceptibility to TB, with epidemiological data indicating 2-3 times higher TB incidence and up to four times higher mortality than younger patients. In several East Asian regions, including Japan, Korea, and Hong Kong, older adults accounted for 45-69% of TB cases in 2020-21 [4]. Similarly, projections from Taiwan predict 78% of new TB cases by 2035 will arise in the geriatric population, which currently contributes to 82.1% of TB-associated mortality [5, 6]. These trends underscore the urgent need to elucidate age-associated immune defects driving TB vulnerability in an aging world.

Despite this growing burden, current TB research predominantly employs young adult mouse models (6-8 weeks old, equivalent to ~18-year-old humans), which do not adequately capture the immune landscape of older hosts [7]. Evidence from studies suggests that old (>18 months; M) mice exhibit higher bacterial burden, delayed CD4^+^ T cell responses, and altered immune activation compared to younger counterparts [8, 9, 10]. However, the impact of immunosenescence on bacterial clearance dynamics, immune cell phenotypes, and host responses during TB treatment remain largely unexplored.

To address this knowledge gap, we investigated age-dependent effects on Mtb infection and treatment in C57BL/6 mice spanning young (2-4M), mid-aged (9-12M; 38-44 human years), old (17-19M; ~54-60 human years), and aged (31M; >80 human years) stages. Following low-dose aerosol infection with Mtb H37Rv and treatment with rifampicin and isoniazid (RIF-INH), we monitored bacterial clearance kinetics across tissues, profiled systemic and tissue-specific immune responses, including circulating cytokines, Mtb-specific antibodies, liver micronutrients, and the proteome of splenic CD4^+^CD44^+^ T cells. Through this approach, we aimed to identify age-associated processes that compromise treatment efficacy, which can inform strategies to reduce the risk of relapse in geriatric TB patients.

## RESULTS

### Lung Mtb burden in C57BL/6 mice remains unaffected by age or sex through 6 weeks post-infection

The influence of host age and sex on susceptibility to Mtb infection remains an area of interest, particularly at early time points. Thus, female and male C57BL/6 mice of wider age groups were aerosol infected with a low dose of H37Rv (Figure 1A). Mice were categorized as young (2-4M), middle-aged (9M females, 12M males) and old (17-19M males). Prior to Mtb infection, body weight was higher in middle-aged and older mice than in younger controls (Supplementary Figure 1). During the study period, old mice lost ~3% weight, whereas young mice gained ~8% weight (Figure 1B). Young female mice gained more body weight (~20%) than males (~10%) (Figure 1B). At 6 weeks post infection (w.p.i.), middle-aged male mice showed significant body weight loss (Figure 1B). Despite age-related variations in body weight, lung Mtb burden progressively increased up to 4 w.p.i. before plateauing by 6 w.p.i., with no significant differences attributable to age or sex (Figure 1C). Thus, immunosenescence manifests primarily during therapeutic clearance rather than initial containment phase, rationalizing our focus on age-associated differences in treatment response.

**Figure 1:**
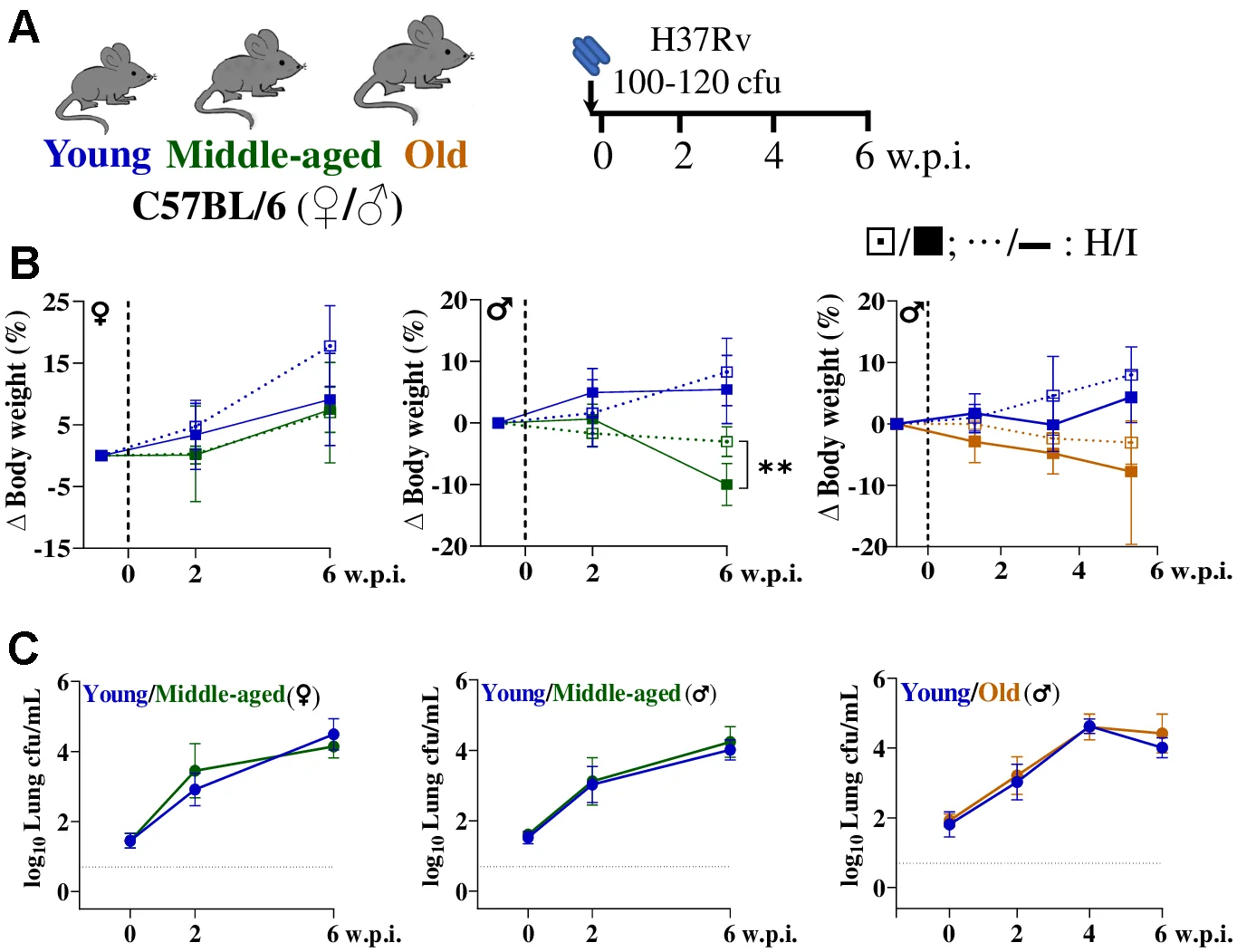
**Lung Mtb burden remains similar till 6 weeks post-infection across age groups and sexes in C57BL/6 mice.** (**A**) Schematic of the experimental design used for aerosol Mtb H37Rv infection of C57BL/6 mice [female (**♀**) and male (**♂**)] across age groups. (**B**) Changes in body weight of mice groups (healthy: H, ^….^; infected: I, ^___^) are presented, n=5-10/age group/condition; dashed vertical line represents day of infection. (**C**) Lung bacterial burden (in log_10_cfu/mL) in mice post Mtb infection; n=5-10/timepoint/age group/condition; dashed horizontal line represents limit of detection (LOD). Young (2-4 months) in blue, middle-aged (9-12 months) in green and old (17-19 months) mice in brown; cfu= colony forming unit; w.p.i.= weeks post infection; p-value: ** <0.005 at 95% confidence interval by Mann-Whitney test. Data shown as mean ± SD. See also Supplementary Figures 1, 2.

### Inflammaging and immune defects drive delayed TB clearance in aging despite hepatic resilience

To evaluate the impact of age on treatment response, a subset of mice received RIF-INH following Mtb infection. Remarkably, older mice retained a significantly higher ~3 log_10_ cfu lung Mtb load at 2 weeks post-treatment (w.p.t.) compared to the young group (~1.5 log_10_ cfu). And by 4 weeks, both mouse age groups achieved complete bacterial clearance, i.e. resulting below the limit of detection (LOD) (Figure 2A, 2B). Histologically, old mice developed granulomatous lung lesions with prominent foamy cells later (6 w.p.i.) compared to young mice (4 w.p.i.) (Supplementary Figure 1). Lung inflammation was resolved in young mice by 4 w.p.t. but persisted in old mice (Supplementary Figure 1). Additionally, middle-aged mice were able to resolve the inflammation by 4 w.p.t., irrespective of sex (Supplementary Figure 2). In extrapulmonary sites, such as spleen and liver, mycobacterial clearance was observed by 2 w.p.t. in both age groups (Figure 2B).

**Figure 2:**
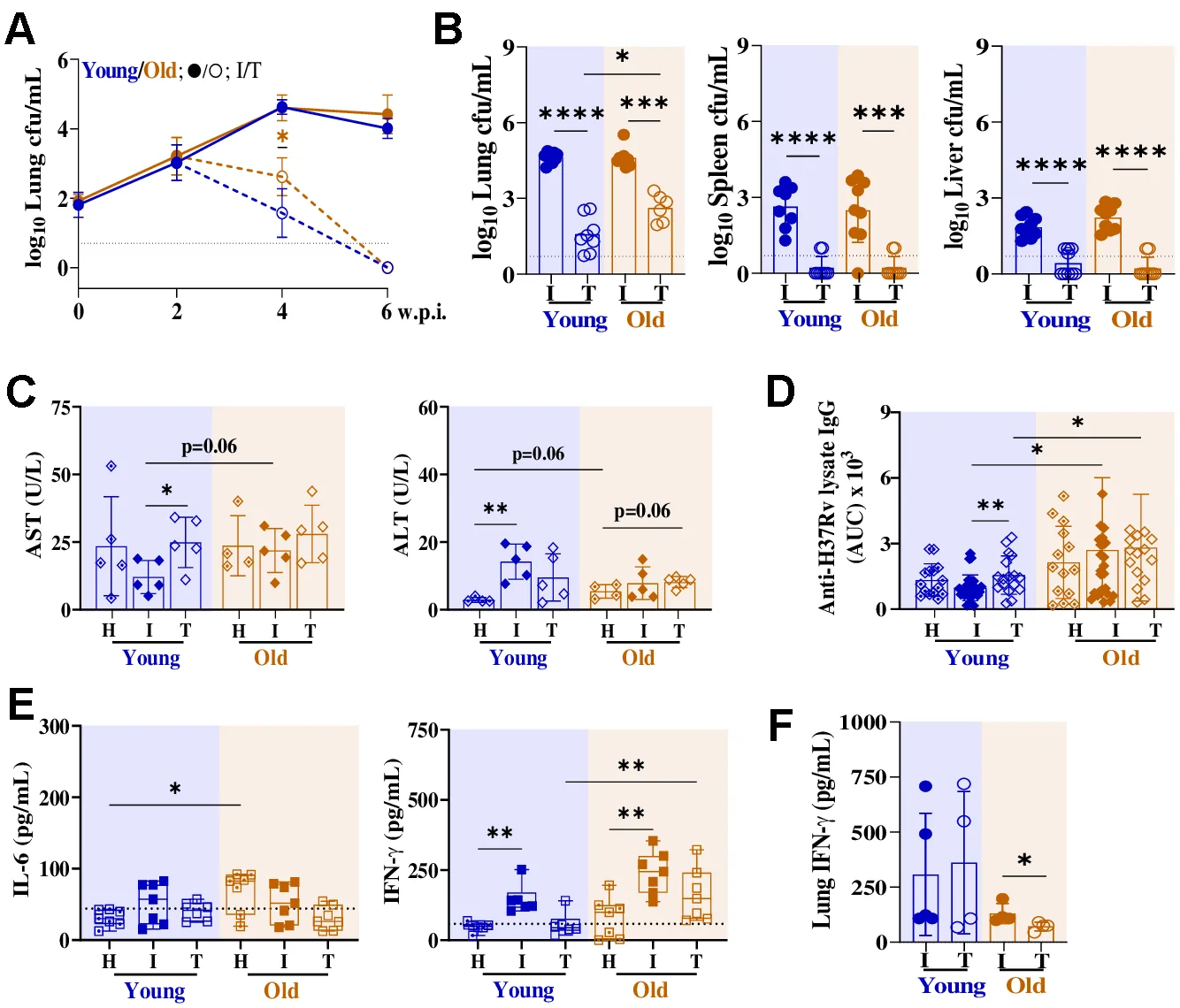
**Old C57BL/6 mice exhibit delayed lung Mtb clearance following two weeks of RIF-INH treatment.** (**A**) Lung bacterial burden (in log_10_cfu/mL) in male C57BL/6 mice from infected (I; at 2, 4 and 6 w.p.i.) and treated (T; at 2 and 4 w.p.t.; treatment started at 2 w.p.i.) groups; n=10/timepoint/age group/condition; dashed horizontal line represents limit of detection (LOD). A set of two independent experiments is depicted. (**B**–**F**) showing data of infected groups at 4 w.p.i. and treated groups at 2 w.p.t.. (**B**) Bacterial load (in log_10_cfu/mL) in lung, spleen and liver; individual data points at 4 w.p.i./2 w.p.t. presented in histograms (n=10/age group/condition). (**C**) Serum alanine transaminase (ALT) and aspartate aminotransferase (AST) activities in mice of different groups (n=5/age group/condition) at 4 w.p.i./2 w.p.t.. (**D**) Area under the curve (AUC) for Mtb H37Rv lysate-specific serum IgG in healthy (H; n=15/age group), infected (I; n=27-28/age group) and treated (T; n= 16-17/age group) mice at 4 w.p.i./2 w.p.t.. (**E**) Serum cytokines (in picogram per milliliter): IL-6, IFN-γ in different mice groups (n=6-7/age group/condition) at 4 w.p.i./2 w.p.t.. (**F**) Lung IFN-γ in the different mice groups (n=4-5/age group/condition) at 4 w.p.i./2 w.p.t.. Young (2-4 months) in blue and old (17-19 months) mice in brown; cfu= colony forming unit; w.p.i.= weeks post infection; w.p.t.= weeks post treatment; p-values: * ≤0.05, ** <0.005, *** <0.0005 and **** <0.0001 at 95% confidence interval by Mann-Whitney test. Data shown as mean ± SD. See also Supplementary Figures 1, 4.

Liver enzymes are pivotal for drug metabolism, with metal ions exerting substantial influence over their activity. Age-related changes may influence this variability in liver function and micronutrient status [11]. Thus, we monitored the liver micronutrient levels of these mouse groups using inductively coupled plasma mass spectrometry (ICP-MS) (Supplementary Figure 3). Old mice had higher hepatic Cu (4.28×), low Zn (0.14×) levels with similar Fe and Se levels compared to controls (Supplementary Figure 3). Additionally, old mice receiving treatment showed higher hepatic Cu levels (Supplementary Figure 3). Liver micronutrient status impacts activities of hepatic enzymes like alanine aminotransferase (ALT) and aspartate aminotransferase (AST), commonly used markers of liver function and injury. Old mice exhibited comparable serum ALT and AST activities to the young mice (Figure 2C). However, ALT activity showed a non-significant upward (p=0.06) trend in old mice (Figure 2C). Mtb infection did not impact serum AST or ALT activity in old mice, but ALT activity was higher in Mtb-infected younger mice (Figure 2C). Strikingly, young mice receiving treatment had higher serum AST activity reflecting drug-induced liver injury, while old mice remained unaffected (Figure 2C).

To assess age-related differences in humoral immunity against Mtb, if any, serum anti-H37Rv specific IgG titers were measured. The overall IgG response was significantly higher in old mice compared to young mice (Figure 2D). Importantly, old mice receiving treatment mounted higher IgG responses at a later time point i.e. 4 w.p.t., while young mice showed it at 2 w.p.t. compared to their respective infected controls (Figure 2D and Supplementary Figure 4). While robust humoral responses are beneficial for pathogen control, the delayed yet heightened antibody response in old mice could indicate persistent antigenic stimulation and likely reflect altered immune regulation with aging. Circulatory IL-6 levels were higher in old mice, consistent with a state of chronic low-grade inflammation or inflammaging (Figure 2E). Higher circulatory IFN**-γ** levels were observed in Mtb-infected young and old mice, which resolved partially in treated groups (Figure 2E). The cytokine response in old mice appears skewed toward a T_H_1-type response rather than a broad systemic inflammatory state, which may explain selective upregulation of IFN-γ without a parallel increase in IL-6. However, at the site of infection, IFN-γ levels trended higher in young mice compared to old mice (Figure 2F). Critically, despite RIF-INH treatment selectively reducing lung IFN-γ levels in old mice, yet bacterial clearance remained impaired (Figure 2F). This reveals a central paradox showing T_H_1 contraction coupled with effector dysfunction, where diminished IFN-γ, rather than unresolved inflammation, impairs bacterial killing during early therapy.

### Age-related T cell dysregulation impairs immune Mtb control

CD4^+^ T cells control Mtb primarily by producing cytokines and licensing infected phagocytes, but aging drives progressive dysfunction marked by exhaustion-like phenotypes. T cells were profiled using activation markers (CD44, PD1), homing receptor (CXCR5), senescence (KLRG1, CD153) and lineage-defining transcription factors (Tbet for T_H_1 and FoxP3 for T_reg_) and a significant change in their distribution was observed in old mice (Supplementary Figure 5). CD44hi marks antigen-experienced CD4^+^ T cells, while PD-1 captures chronically stimulated cells that can be either functional (protective, CXCR5^+^ T_FH_-like) or dysfunctional depending on expression of other markers. CXCR5^+^CD4^+^ T cells support B cell help and are associated with better Mtb control when present in organized structures [12, 13]. KLRG1 is enriched on highly differentiated, senescent-like CD4^+^ T cells that accumulate with age. KLRG1^+^PD1^+^ T represents late-differentiated, senescent-like cells with constrained effector function. Notably, high expression of CD44, CXCR5, PD1 and CD153 in CD4^+^ and CD8^+^ T cells of old mice were observed (Supplementary Figure 5, 6). Increased circulatory CD4^+^KLRG1^+^PD1^+^ cells were observed in old mice, indicative of a higher frequency of exhausted T cell phenotype (Supplementary Figure 5).

Next, we monitored the immune cell distribution in lungs and spleen of Mtb-infected and RIF-INH treated mice groups (Figure 3A, 3B and Supplementary Figure 7). Old mice showed a lower proportion of splenic CD4^+^, higher CD8^+^ T cells, along with higher CXCR5 and PD1 expression compared to young mice (Figure 3C and Supplementary Figure 7). Interestingly, lungs of old mice showed accumulation of CD4^+^CD44^+^CXCR5^+^ (T_FH_)-like cells, similar to young mice post Mtb infection (Supplementary Figure 8). Additionally, CD8^+^CD44^+^CXCR5^+^ (T_FC_)-like cells were observed to be more abundant in the lungs and spleens of Mtb-infected old mice at 4 w.p.i. (Figure 3C and Supplementary Figures 7, 8). T_FC_-like cells are a follicular, B-cell interacting CD8 subset, which compete with T_FH_ cells for germinal centre reactions, disrupting antibody maturation.

**Figure 3:**
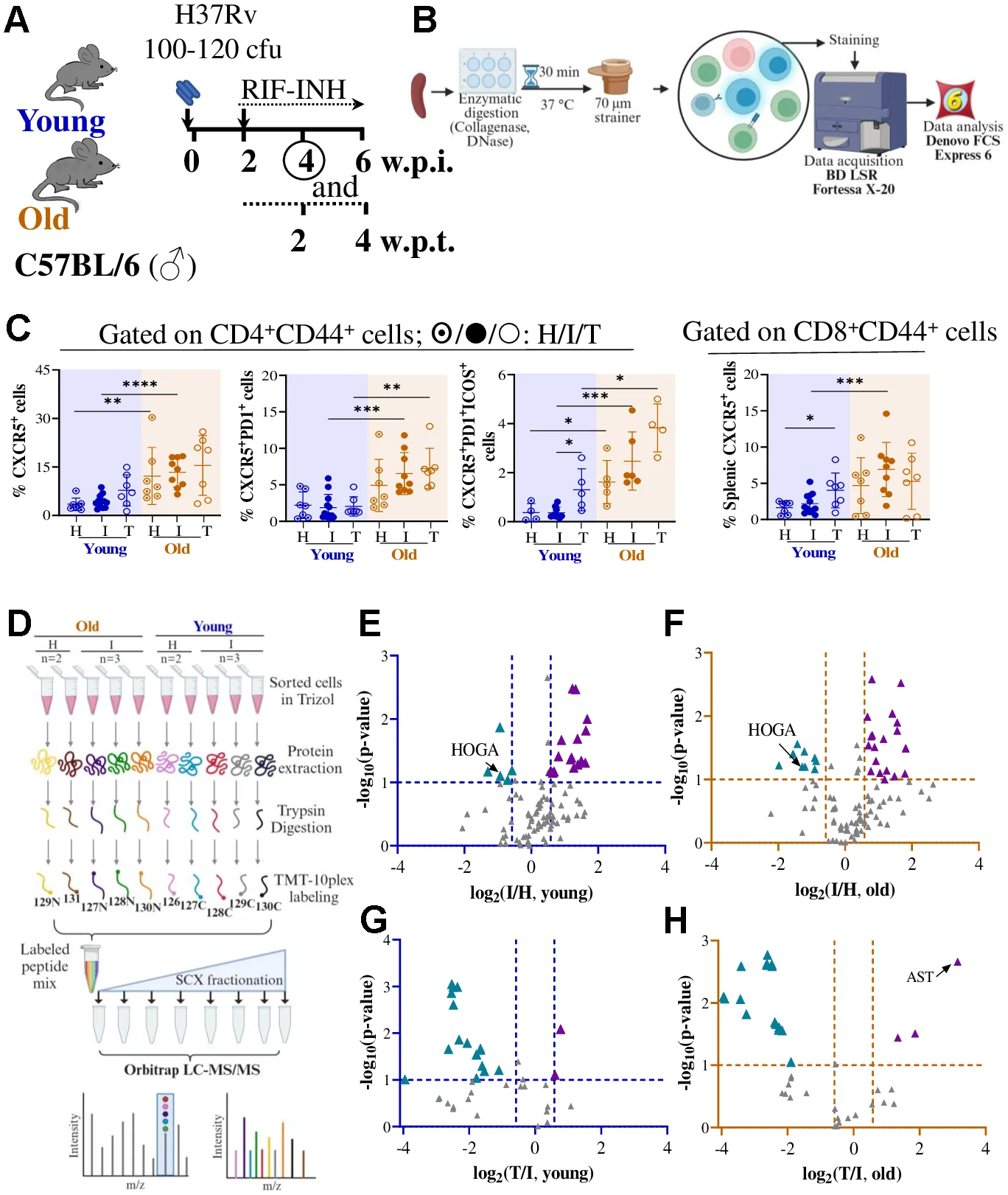
**Deregulated splenic CD4^+^CD44^+^ T cell proteome in old C57BL/6 mice upon Mtb infection and RIF-INH treatment.** (**A**) Schematic showing Mtb H37Rv aerosol infection in male C57BL/6 mice of two age groups (young: 2-4 and old: 17-19 in months) followed by treatment with RIF-INH starting at 2 w.p.i.. (**B**) Workflow for immune cell isolation from the spleen, flow cytometry data acquisition and data analysis. (**C**) Frequencies of CD4^+^CD44^+^CXCR5^+^, CD4^+^CD44^+^CXCR5^+^PD1^+^ and CD4^+^CD44^+^CXCR5^+^PD1^+^ICOS^+^ and CD8^+^CD44^+^CXCR5^+^ cells in the spleen of C57BL/6 mice at 4 w.p.i./2 w.p.t.. Healthy (H; n=3/age group), infected (I; n=6-10/age group) and treated (T; n=3/age group). (**D**) TMT10plex workflow for CD4^+^CD44^+^ T cell proteome analysis (for TMT set-1) by liquid chromatography- mass spectrometry (LC-MS/MS). (**E**–**H**) showing volcano plots of splenic CD4^+^CD44^+^ T cell proteome showing significantly deregulated proteins (in -log_10_p-value ≥ 1; log_2_fold change > |0.58|): (**E**) Young infected versus healthy, (**F**) Old infected versus healthy, (**G**) Young treated versus infected and (**H**) Old treated versus infected; ▲/▲: up-/down-regulation; HOGA: 4-hydroxy-2-oxoglutarate aldolase; AST: aspartate aminotransferase. Young (2-4 months) mice in blue and old (17-19 months) mice in brown; cfu= colony forming unit; w.p.i.= weeks post infection; p-values: * ≤0.05, ** <0.005, *** <0.0005 and **** <0.0001 at 95% confidence interval by Mann-Whitney test. Data shown as mean ± SD. See also Supplementary Figures 7–10 and Supplementary Tables 1–4.

In a separate experimental set, at 4 w.p.i., lung Mtb load was found to be similar between young (2M) and aged (31M) male C57BL/6 mice groups (Supplementary Figure 9). Old and aged mice had overall higher splenic CD4^+^CD44^+^CXCR5^+^PD1^+^, CD4^+^CD44^+^CXCR5^+^PD1^+^ICOS^+^ and CD8^+^CD44^+^CXCR5^+^PD1^+^ cells compared to young mice, irrespective of infection and treatment status (Figure 3C and Supplementary Figure 9). The detailed distribution of immune cells observed in multiple tissues of 2-, 4-, 17-, 19-, and 31M old mice groups is tabulated in Supplementary Table 1. Immunosenescence creates pathological T_FH_-T_FC_ competition, diverting adaptive immunity toward dysregulated CD8^+^ follicular responses that compromise TB therapeutic efficacy.

### Splenic T cell proteome reveals age-specific mitochondrial collapse

To further elucidate the molecular drivers of delayed Mtb clearance, we conducted global proteomic profiling of flow-sorted CD4^+^CD44^+^ T cells (Figure 3D and Supplementary Table 2). In set-I, 109 proteins were identified, and Mtb-infected young and old mice showed 23 and 30 deregulated (p ≤ 0.1, log_2_fold change (log_2_fc): I/H > |0.58|) proteins, respectively (Figure 3E, 3F and Supplementary Figure 10 and Supplementary Table 3). In set-II, out of 41 identified proteins, ~20 were deregulated in the mouse groups receiving RIF-INH treatment (Figure 3G, 3H and Supplementary Table 4). Proteins involved in immune response (e.g., complement, serotransferrin) were suppressed in old mice, potentially reflecting age-related immune decline. Young mice exhibited few changes, with upregulation of stress response proteins like galectin-1 and superoxide dismutase, possibly reflecting a more robust acute response (Supplementary Table 3). Both mouse age groups showed downregulation of 4-hydroxy-2-oxoglutarate aldolase (HOGA), suggesting disrupted hydroxyproline degradation [14] (Supplementary Table 3). Upon Mtb infection, old mice showed an upregulation of prostaglandin E synthase 3 (PTGES3, log_2_fc: I/H = 1.15), indicating an age-biased induction of prostaglandin synthase E2 (PGE2) machinery (Supplementary Table 3). PGE2 disrupts alveolar macrophage metabolic fitness through mitochondrial dysfunction, suppressing electron transport chain gene expression [15].

Additionally, old mice showed elevated levels of mitochondrial AST after treatment (log_2_fc: T/I = 3.15), indicative of unresolved mitochondrial stress, consistent with increased serum AST and altered hepatic micronutrient status observed earlier (Supplementary Table 4). Old mice showed lower HOGA abundance after treatment, implying prolonged mitochondrial stress in T cells of old mice (Supplementary Table 4). Together, these proteomic signatures reveal age-specific mitochondrial defects, providing a molecular basis for T cell dysfunction, inflammaging, and impaired mycobacterial clearance in older hosts, linking cellular metabolic stress in T cells during Mtb infection and therapy.

## DISCUSSION

As the global demography is shifting towards the older (≥60 years) age groups, it is expected that immunosenescence and lack of an effective TB vaccine for adults will be key drivers for the elevated numbers of geriatric TB patients. Paradoxically, in countries where TB was contained successfully, the number of new TB cases is increasing, for example, in Kansas City and North Carolina in the United States. The majority of published reports focused on understanding host-pathogen interactions in Mtb-infected mice of 6-8 weeks (~2M), representing a human age equivalence of 18-20 years [7]. Thus, understanding how the older host responds to Mtb infection is crucial for developing appropriate and effective interventions.

TB demonstrates a marked gender disparity, with males showing increased susceptibility, with male to female ratio of 1.7 in TB prevalence, which is associated with reduced B cell follicle formation in the lung [16–18]. On the contrary, females exhibit increased T cell activity, production of T_H_1-type cytokines, particularly IFN-γ, and robust antibody responses [19]. Above 65 years of age, genomic disparities between the sexes become more pronounced, with males exhibiting higher innate and pro-inflammatory activity [20]. Thus, we focused on using old male mice to study the outcomes upon Mtb infection and post-treatment.

A low aerosol dose of Mtb infection to young (2-4M), middle-aged (9, 12M), old (17-19M) and aged (31M) C57BL/6 mice yielded similar lung mycobacterial burden up to 6 w.p.i.. Whereas earlier reports showed better Mtb control in older mice at earlier time points [8, 9, 21, 22]. Turner et al. reported that 18M C57BL/6 mice aerosol-infected with Erdman strain had a lower lung load at 14 d.p.i., compared to 3M controls [9]. 24M B6D2F1 hybrid mice infected with H37Rv showed lower load at 20 d.p.i. than young 2M controls [8]. Variations in reported tissue Mtb loads between earlier reports and this study may stem from differences in mouse strain and sex, Mtb strain and dose, infection method, and observation timing. Important to highlight that, to the best of our knowledge, this report demonstrates for the first time, that old mice showed a compromised Mtb clearance after 2 weeks of RIF-INH treatment compared to younger controls. These results corroborate the reported delayed viral clearance in 17M BALB/c mice infected with PR8 influenza virus [23].

Aging also impacts hepatic function, affecting the metabolism of exogenous molecules, thereby leading to hepatoxicity [24]. Old mice displayed a higher hepatic Cu/Zn ratio irrespective of infection status, consistent with reports that an elevated Cu/Zn ratio in aged hosts is a biomarker of chronic inflammation and oxidative stress. The accumulation of Cu and Zn in phagosomes increases bactericidal activity; however, Cu overload is detrimental to the host [25]. Low Zn levels in older mice may impair Cu/Zn superoxide-dismutase function, leading to pro-inflammatory effects and weakened hepatic function [26]. Mtb pathogenesis depletes the available Fe pool and is a typical host response in counteracting Mtb replication in TB patients [27]. The liver Fe level was reduced in young mice post-treatment; however, treated old mice retained a higher hepatic Fe pool, providing a niche that could support Mtb growth and persistence. Dysregulated iron homeostasis could influence bacterial burden partly through effects on ferroptosis, which is an important avenue for future mechanistic work. Hepatic micronutrient imbalances in old mice did not translate to significant serum ALT/AST elevations or drug-induced liver injury, unlike in young treated mice. This hepatic resilience underscores that age-related treatment delays stem from immune defects rather than metabolic or toxic limitations. Young mice maintained high lung IFN-γ to drive rapid bacterial killing while old mice exhibited premature T_H_1 contraction, creating a confluence of impairments during early-phase treatment.

Immune cells, such as CD4^+^ T cells play a critical role in controlling Mtb, and their function might be partially impaired in older hosts [28]. A higher lung mycobacterial load was predicted in 18M mice at 6 w.p.i. compared to 3M mice, due to a delayed CD4^+^ T cell response [28, 29]. We observed that Mtb-infected old C57BL/6 mice had similar T_FH_-like cells in the lungs and a higher frequency in the spleen at 4 w.p.i. compared to younger controls. However, a higher proportion of T_FC_-like cells was observed in the lungs and spleens in Mtb-infected old mice. The contribution of T_H_1 cells in TB is well established. However, our understanding of T_FH_ cells, which link humoral and adaptive immunity, during Mtb infection is limited [30]. T_FC_ cells suppress T_FH_ cell helper function and antibody responses through multiple mechanisms, thereby inhibiting antibody production to maintain self-tolerance and regulate immune responses [31, 32]. Age is reported to negatively influence T_FH_ cell function and differentiation, thereby compromising host responses [33]. Localization of T_FH_-like cells has been demonstrated within protective granuloma-associated lymphoid tissue in lungs of 2M C57BL/6 mice, which mediates Mtb control [34]. In effective responses, T_FH_ cells dominate the follicular niche, provide IL-21 and CD40L to B cells, and help structure organized germinal centres or tertiary lymphoid-like granulomas. Immunosenescence drives this pathological skew: aging-associated T_FH_ cells enable T_FC_ expansion, disrupting germinal centre formation and granuloma organization.

Understanding tissue-level T cell distribution in Mtb-infected old mice has been attempted earlier; however, limited reports captured the changes during TB treatment [8, 9, 21, 28, 30]. At 2 w.p.t., young mice showed increased splenic T_FH_-like cells, which remained similar in old mice, suggesting the inability of the older host to elicit a proper T_FH_ response. Compromised Mtb clearance in old mice might be due to the impaired T_FH_ cell functionality, which was reported earlier in >20M old C57BL/6 mice [12, 33]. Future work should phenotypically characterize T_FC_-like cells to test their active suppression of protective T_FH_ responses, establishing causality in age-dependent humoral dysregulation.

Proteome analyses of splenic cells from Mtb-infected old mice revealed altered abundance of mitochondrial enzymes involved in the catabolism of hydroxyproline (Hyp), a product of protein degradation, notably higher AST and lower HOGA [14] (Figure 4). AST catalyzes 4-hydroxy glutamate (4-OH-Glu) to 4-hydroxy-2-oxo-glutarate (HOG), which is converted into pyruvate and glyoxylate using HOGA. This imbalance likely causes HOG accumulation, inhibiting glyoxylate reductase and promoting excessive oxalate production, known to disrupt mitochondrial function independent of the lactate dehydrogenase pathway [35]. PTGES3 upregulation in Mtb-infected old mice further implicates prostaglandin E2 signaling, which prior work shows hampers alveolar macrophage proliferation and mitochondrial function in 18-22M mice [15]. These proteomic changes represent immunometabolic signatures of T cell dysfunction in Mtb-infected old mice rather than as direct causal drivers. The observed HOGA downregulation and PTGES3 signaling likely underlies defective CD4^+^ T cell responses and impaired Mtb clearance. This aligns with evidence that mitochondrial rescue strategies enhance TB immunity in older adults, positioning mitochondrial axis as a therapeutically actionable target [36, 37].

**Figure 4:**
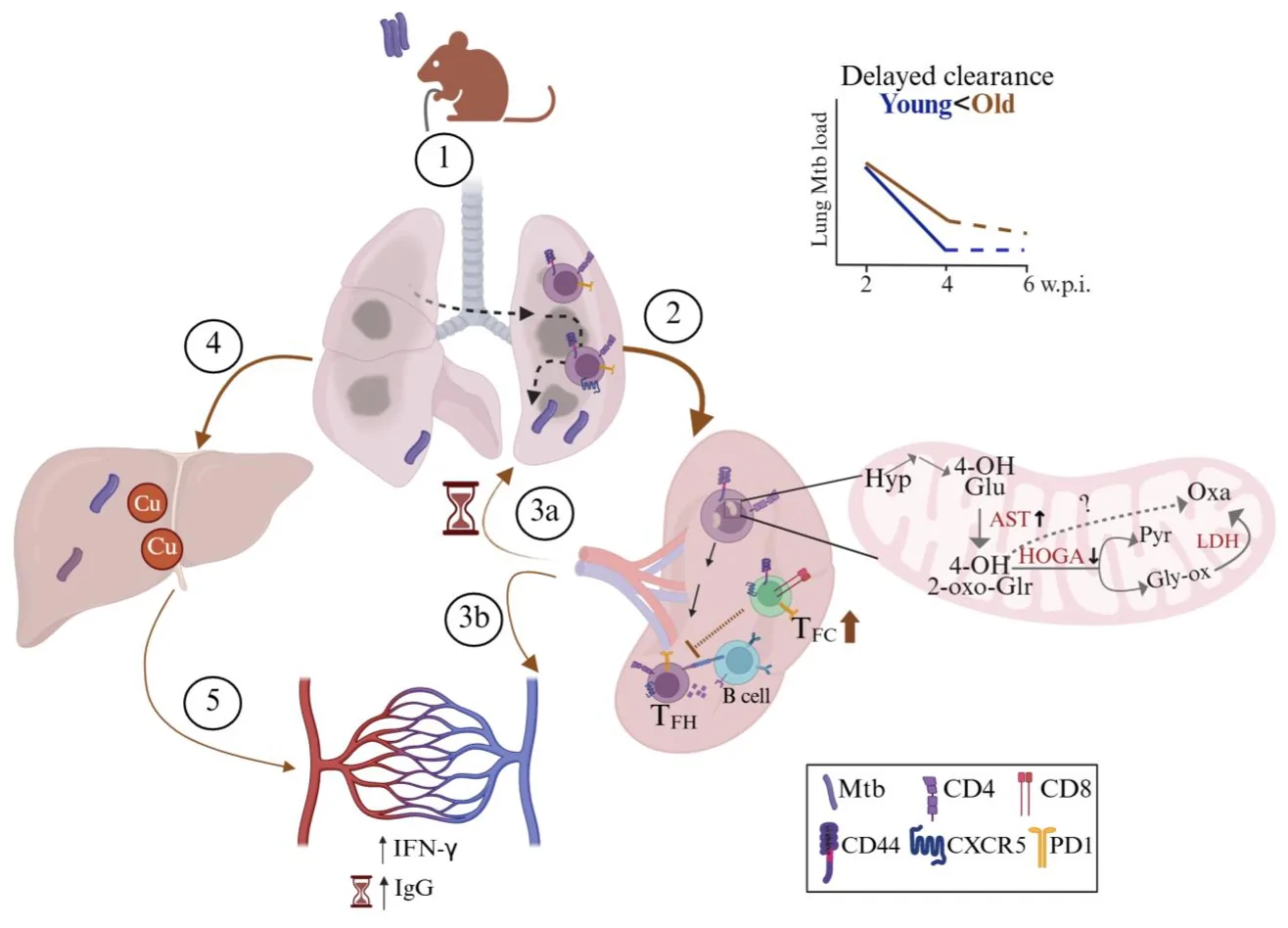
**Age-associated mechanisms of delayed *Mycobacterium tuberculosis* lung clearance in old C57BL/6 mice during early rifampicin-isoniazid treatment.** Illustrative summary of the results observed in the study. (1) Following Mtb infection via aerosol challenge, (2) antigen presentation takes place in the spleen. (3a) T cells reach the inflamed lung of old mice. (3b) Increased levels of proinflammatory cytokines: interferon-gamma (IFN-γ) and increased Mtb-specific IgG levels observed at later timepoint in Mtb-infected old mice. (4) Mtb disseminates to the liver via hepatic artery where copper (Cu) was observed to be accumulated (5) alongside increased systemic proinflammatory mediators. Old mice exhibit dysfunctional splenic T follicular helper (T_FH_ : CD4^+^CD44^+^CXCR5^+^PD1^+^) cells as a virtue of high T follicular cytotoxic cells (T_FC_ : CD8^+^CD44^+^CXCR5^+^PD1^+^), and leads to delayed lung Mtb clearance at 2 weeks post-treatment. Proteome analysis revealed splenic CD4^+^CD44^+^ T cells of old mice showed alterations in mitochondrial proteins (decreased HOGA: 4-hydroxy-2-oxoglutarate aldolase and increased AST), disrupting hydroxyproline (Hyp) degradation and promoting 4-hydroxy-2-oxo-glutarate (4-OH-2-oxo-Glr) accumulation, directing the reaction to oxalate (Oxa) via a pathway independent of lactate dehydrogenase (LDH), pyruvate (Pyr) and glyoxylate (Gly-ox). Created with BioRender.

This study has a few limitations. Technical constraints prevented direct proteomic profiling of lung T_FH_ cells, necessitating analysis of splenic CD4^+^CD44^+^ T cells as a functional proxy. Mouse models in TB research have limitations for extrapolating findings to humans due to differences in disease pathology, notably the absence of key features such as granuloma liquefaction, cavitation, and fibrosis. Also, mice exhibit different metabolic and aging patterns compared to humans, with distinct changes in organ metabolism, immune responses, and biomarkers of aging. Thus, age-associated metabolic alterations identified should be viewed as hypotheses for mechanisms that may operate in older humans, rather than as directly translatable biomarkers. Functional validation of mitochondrial defects including mitophagy flux assay, Seahorse bioenergetics or electron microscopy, remains a key next step. *In vitro* modulation of HOGA/AST (via inhibitors or overexpression) in T cells of lungs would test causality in rescuing anti-Mtb function. Future directions extend beyond T cell-intrinsic changes to dendritic cell antigen presentation and alterations in myeloid cell populations, and using clinical Mtb isolates in aging models for enhanced translational relevance.

In summary, this study demonstrated impaired pulmonary Mtb clearance in old C57BL/6 mice during RIF-INH treatment, driven by a confluence of immunosenescence defects: inflammaging, delayed T_H_1 responses, higher T_FC_ cell frequency and mitochondrial proteome collapse. These findings illuminate age-specific mechanisms contributing to suboptimal TB treatment responses in geriatric patients and identifying novel mitochondrial targets for adjunct therapy.

## MATERIALS AND METHODS

### Experimental groups, Mtb infection and treatment

The procedures adopted in this study were performed following the recommended Guidelines for Care and Use of Laboratory Animals and approved by the Animal Ethics Committee of ICGEB- New Delhi (Ref No. ICGEB/IAEC/07032020/TH-10). C57BL/6 mice of different age groups (2-, 4-, 9-, 12-, 17-, 19- and 31 months, M) were procured from the ICGEB bio-experimentation facility. The mice age equivalents to humans, used in this study, is as follows: young (2-4M), mid-aged (9-12M; 38-44 human years), old (17-19M; ~54-60 human years), and aged (31M; >80 human years) [7]. These mice were transferred to the BSL-III Tuberculosis Aerosol Challenge Facility (TACF) for experiments involving Mtb infection. Mice were fed a chow diet, water ad libitum and maintained at 20-22° C; 45-60% humidity with a 12h light/dark cycle. Mice were weight-adjusted, randomly grouped and after 1 week of acclimatization in TACF, infected with a low dose (100-120 cfu) of Mtb H37Rv strain in the Madison aerosol exposure chamber. After 2 w.p.i., a subset of mice received RIF and INH (100 mg/L each) in drinking water and fresh drug solutions were replaced every 48 hours. Two 17M mice (one bearing an abscess, the other died before the completion of 4 w.p.t.) were excluded from the study.

### Cfu assay

Tissues (lung, spleen and liver) were homogenized in PBS (1 mL) and plated on 7H11 agar supplemented with Middlebrook 10% OADC. These plates were incubated at 37° C, and bacterial colonies were enumerated 3 weeks later.

### Histopathology

Formalin-fixed lungs were paraffin-embedded, sectioned and stained with hematoxylin and eosin, followed by capturing images using Nikon Eclipse TS2. The slides were scored based on the severity of inflammation, granuloma formation and necrosis.

### Liver micronutrient profiling

Liver (~50 mg) was subjected to microwave digestion post addition of H_2_O_2_ and HNO_3_ (1:4). These were diluted and introduced to iCAP™-TQ ICP-MS using argon as carrier. Data acquisition was performed using Qtegra (Thermo Fisher Scientific, version 2.10.4345.39) and involved running different concentrations of a multielement standard mix (Supelco, #92091).

### ALT and AST assay

Serum ALT and AST activities were measured using kits (Cayman Chemical, #700260 and #701640) according to the manufacturer’s instructions.

### Immune cell isolation and flow cytometry

Harvested tissues (lung and spleen) were incubated with collagenase D (1 μg/mL) and DNase I (0.5 mg/mL). Peripheral blood mononuclear cells (PBMCs) were isolated using Histopaque-1077 following standard procedures. Isolated cells were stained with recommended dilutions by the manufacturer: CD3-BV510 (BioLegend, #100234), CD4-BV785 (BioLegend, #100552), CD8a-AF700 (BioLegend, #100730), CD44-BV605 (BioLegend, #103047), CXCR5-PE/Dazzle 594 BioLegend, #145522), PD1-PE-Cy7 (BioLegend, #109110), KLRG1-BV421 (BioLegend, #138414), CD153-PE (BioLegend, #106405), CD25-AF488 (BioLegend, #102017) and CD127-BV650 (BioLegend, #135043) and LIVE/DEAD™ Fixable Near IR stain (Invitrogen, #L34994) on ice for 45 min. After washing with buffer (0.2% FBS in 1× PBS), cells were treated with fixation (Invitrogen, #00-5123) and permeabilization buffer (Invitrogen, #00-8333) and kept on ice for 30 min. Intracellular staining was done with FoxP3-AF647 (BioLegend, #126408) and Tbet-BV711 (BioLegend, #644820) and incubated at RT for 45 min, followed by washing. Data (~1 million events/sample) was acquired using BD LSRFortessa X-20 and analyzed using FCS Express (DeNovo software, version 6).

Another set of immune cells were stained with: CD3-FITC (Invitrogen, #11-0032-82), CD4-SB600 (Invitrogen, #63-0042-82), CD8a-BV421 (BioLegend, #100753), CD44-BV510 (BioLegend, #103044), CXCR5-PE, ICOS-PerCP/Cy5.5 (BioLegend, #145504), PD1-PE-Cy7 (BioLegend, #109110) and LIVE/DEAD™ Fixable Near IR stain (Invitrogen, #L34994) on ice for 45 min. After washing with buffer (3% FBS in 1× PBS), splenic CD4^+^CD44^+^ T cells were sorted using BD FACS Aria III Fusion. Data was analyzed using FlowJo (BD Biosciences, version 10.8.1).

Lung IFN-γ estimation: IFN-γ was estimated in lung homogenates by using the ELISA MAX™ Standard Set Mouse IFN-γ kit (BioLegend, #430801) following the manufacturer’s instructions.

### Serum cytokine estimation

Two times diluted serum samples were used to estimate cytokines using capture bead-based assay LEGENDplex (BioLegend) along with the standard (BioLegend, #740371, lot no. B330381). The samples were acquired on BD LSRFortressa X-20 using PE and APC as the reporter and bead classification channel, respectively. Data was analyzed using Qognit software.

### Serum IgG antibody quantification

ELISA plate was coated with Mtb H37Rv whole cell lysate (10 μg/mL) and incubated overnight at 4° C. Then, wash buffer (0.05% Tween-20 in 1× PBS) was added, followed by blocking in 3% BSA in 1× PBS containing 0.05% Tween-20 and incubated at 37° C for 2h at 180 rpm. After blotting the plate dry, sera were serially diluted fourfold in 0.1% BSA in 1× PBS containing 0.05% Tween-20, and 100 μL was added per well. The plate was then incubated overnight at 4° C. The plate was washed three times, followed by incubation with anti-mouse IgG (Southern Biotech, #1031-05) at 37° C for 2h at 180 rpm. The plate was washed six times and developed by adding o-phenylenediamine dihydrochloride, incubated at RT for 5 min. The reaction was terminated by adding 1N HCl, and the absorbance was measured at 490 nm.

### Splenic CD4^+^CD44^+^ cell proteome analysis

Proteins were isolated using TRIzol reagent from sorted splenic CD4^+^CD44^+^ cells and quantified using BCA Protein Assay. An equal amount (~10 μg) of protein from each sample in a set (n = 10) was used for TMT10plex (Thermo Fisher Scientific, #90113). TMT-labelled peptides from all samples were pooled, dried and subjected to strong cation exchange chromatography and eluted using ammonium formate. Later, these fractions (n = 5) were cleaned up using C18 spin columns and dried, resuspended in formic acid (0.1%, 20 μL) and injected into an Orbitrap Fusion Lumos Tribrid Mass Spectrometer connected to a nano-LC 1200. Data was analysed with Proteome Discoverer version 2.3 using *Mus musculus* (ID: UP000000589; 55,087 protein count). Proteins qualifying the parameters [log_2_ fold change ≥ ± 0.58; log_10_p-value ≥1; false discovery rate<0.05] were selected as important deregulated molecules.

### Statistical analysis

Data are presented as mean ± standard deviation. Details on the sample size and statistical analysis are provided in the figure legends.

## Supplementary Material

Supplementary Figures

Supplementary Tables 1 and 2

Supplementary Table 3

Supplementary Table 4
