# Supplementary material for: Host immunosenescence compromises Mycobacterium tuberculosis clearance
Source: Aging (Albany NY). 2026 May 4;18(1):397–420. doi: 10.18632/aging.206374 (PMC13632697; doi:10.18632/aging.206374)
Supplement: Supplementary file 1 [file aging-18-1-206374-s001.pdf]

SUPPLEMENTARY FIGURES

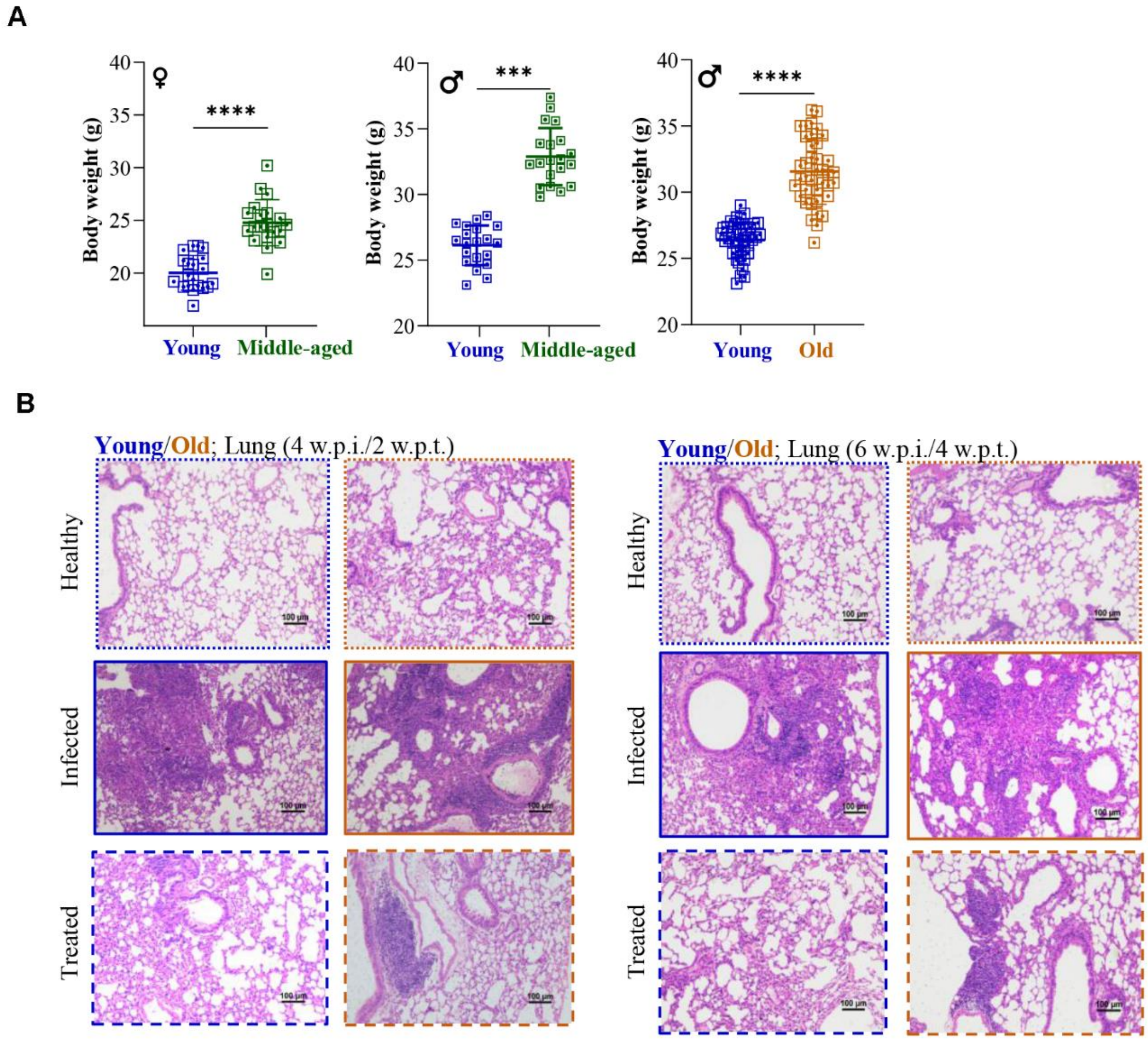

Supplementary Figure 1. Old C57BL/6 mice receiving two weeks of RIF-INH treatment showed delayed lung Mtb clearance. (**A**) Body weight (in gram) of mice (n=20/age group for young and middle-aged panels, n=40/age group for young and old panel) prior to infection (day -5). (**B**) Lung histopathological images (hematoxylin and eosin; H&E stained, 100× magnification, scale bar=100 μm) at 4 w.p.i./2 w.p.t. and 6 w.p.i./4 w.p.t. Young (2-4 months) in blue, middle-aged (9-12 months) in green and old (17-19 months) mice in brown; w.p.i.: weeks post infection; w.p.t.: weeks post treatment; p-values: *** <0.0005 and **** <0.0001 at 95% confidence interval by Mann-Whitney test. Data shown as mean ± SD. *See also Figures 1, 2.*

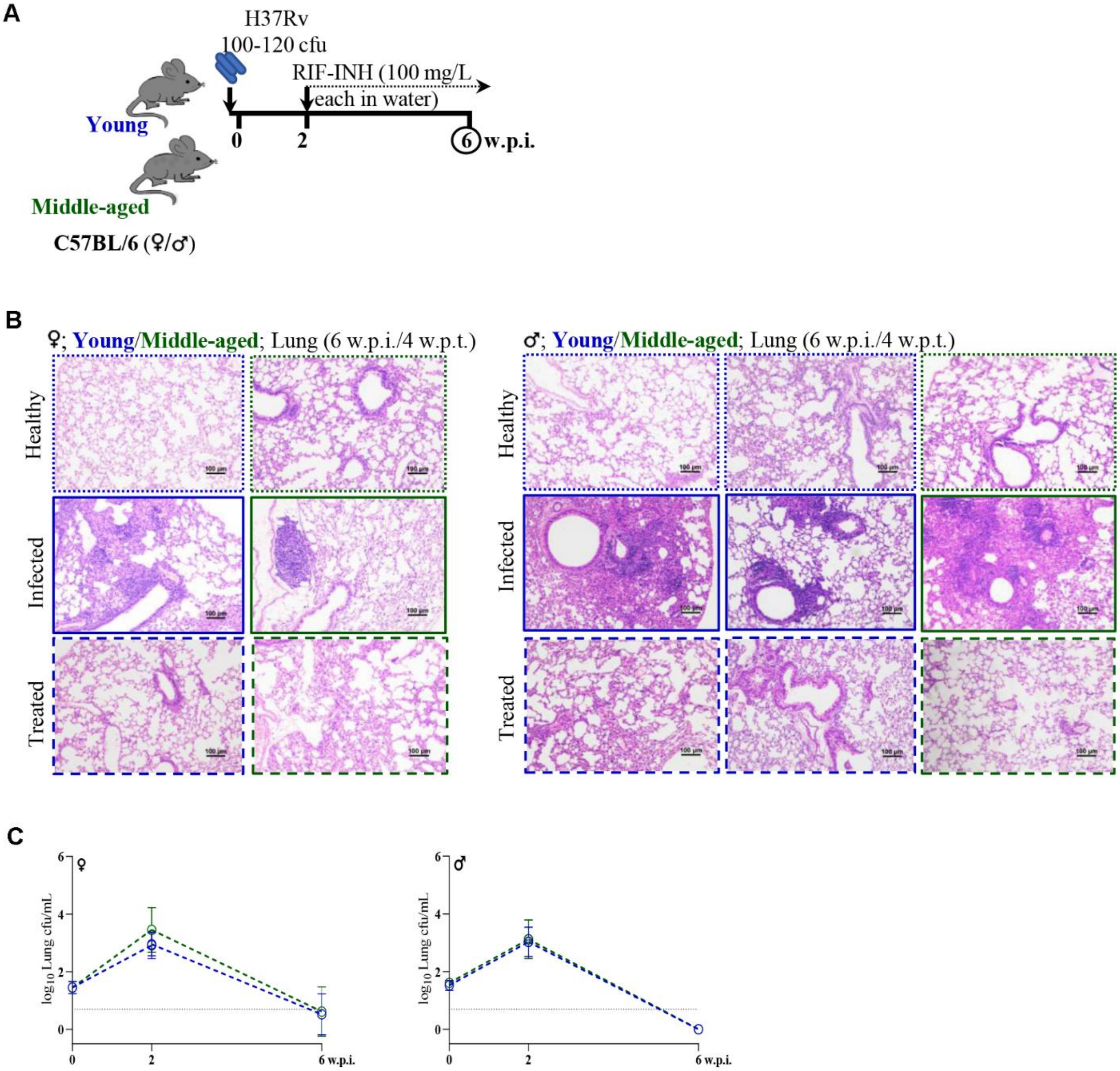

Supplementary Figure 2. Middle-aged C57BL/6 mice showed similar treatment outcomes post Mtb infection compared to younger mice irrespective of gender. (**A**) Schematic of the experimental design for Mtb H37Rv infection and RIF-INH treatment of C57BL/6 mice (female: age groups of 2 and 9 months and male: age groups of 2 and 12 months; M). (**B**) Lung histopathological images (hematoxylin and eosin: H&E stained, 100× magnification, scale bar=100 μm) at 6 w.p.i./4 w.p.t. along with their respective healthy controls. *One set of images of the young mice group at 6 w.p.i./4 w.p.t. was also shown in Supplementary Figure 1B, as a part of the same infection experiment.* (**C**) Lung bacterial burden (in log10cfu/mL) in mice belonging to treated group (at 4 w.p.t.; treatment started at 2 w.p.i.) groups; n=5/timepoint/age group/condition; dashed horizontal line represents limit of detection. Young (2 months) mice in blue and middle-aged (9-12 months) mice in green; cfu: colony forming unit; w.p.i.: weeks post infection; w.p.t.: weeks post treatment. Data shown as mean ± SD. *See also Figure 1.*

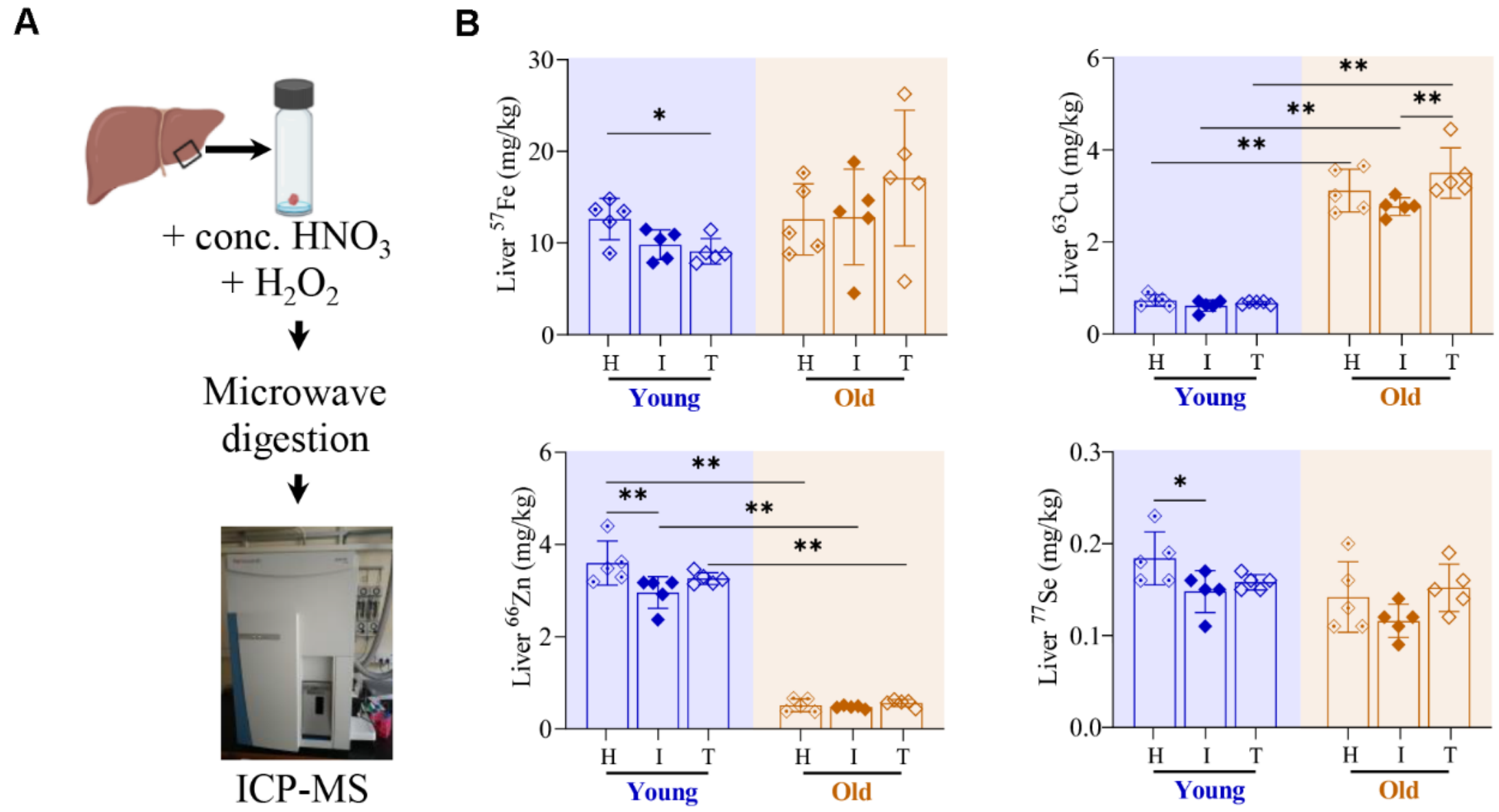

Supplementary Figure 3. Impaired Mtb clearance in old C57BL/6 mice potentially correlates with abnormal levels of liver micronutrients. (**A**) Method adopted for liver micronutrient profiling by inductive coupled plasma mass spectrometry (ICP-MS). (**B**) Abundance of mice liver micronutrients (in milligram per kilogram): iron- ^{57}Fe, copper- ^{63}Cu, zinc- ^{66}Zn, and selenium- ^{77}Se of healthy (H), Mtb H37Rv infected (I) and RIF-INH treated (T) groups at 4 w.p.i./2 w.p.t.; n=5/age group/condition. p-values: * ≤0.05 and ** <0.005 at 95% confidence interval by Mann- Whitney test. Data shown as mean ± SD. Young (2 months) in blue and old (17 months) mice in brown.

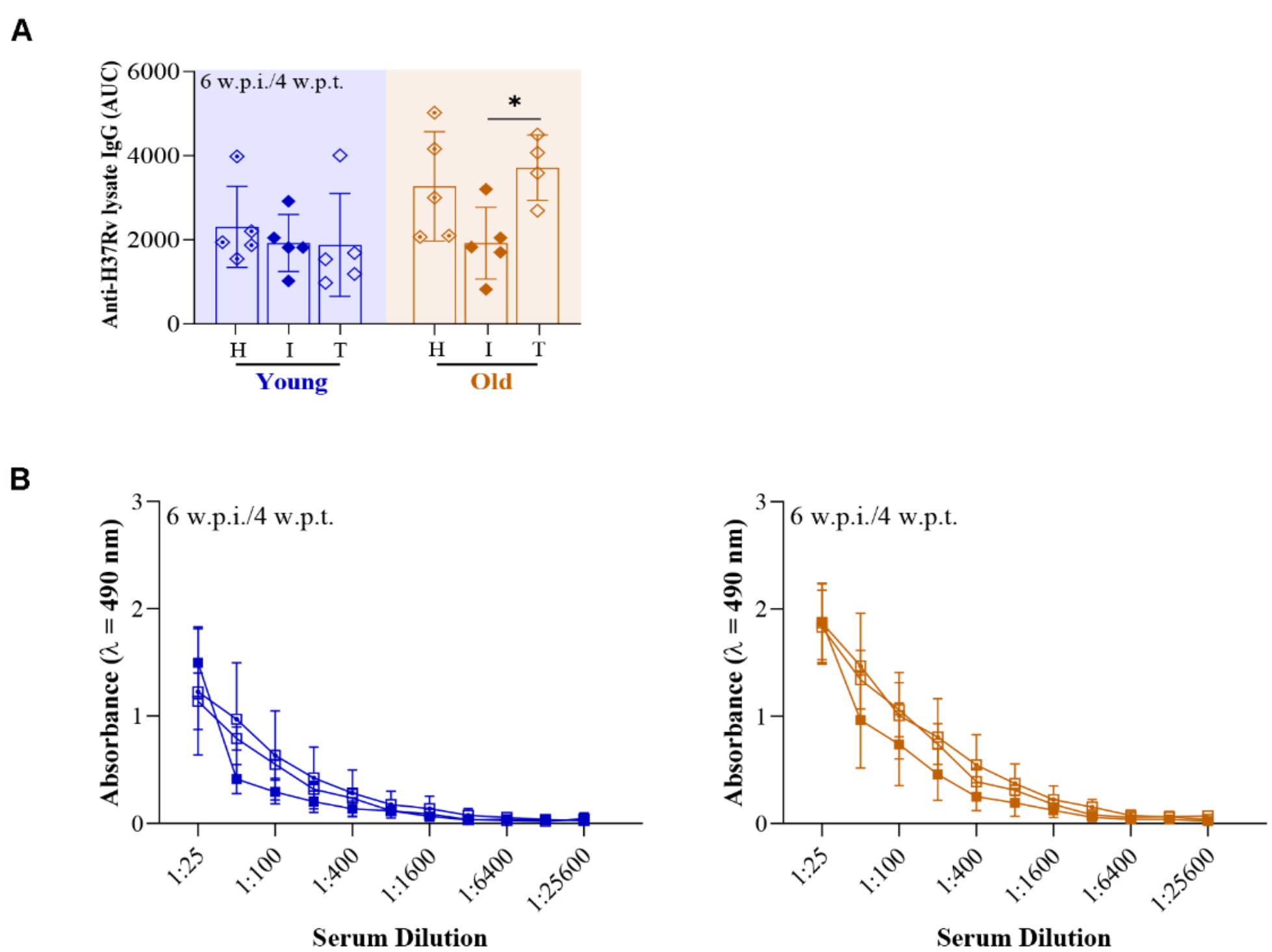

Supplementary Figure 4. Serum anti-Mtb IgG was higher in old mice after RIF-INH treatment. (**A**) Anti-H37Rv lysate IgG estimation with area under the curve (AUC) in the circulation of healthy (H), Mtb H37Rv infected (I) and RIF-INH treated (T) C57BL/6 mice at 6 w.p.i./4 w.p.t.. (**B**) End-point serum dilutions to monitor anti-IgG titers in mice groups at 6 w.p.i./4 w.p.t.; n = 5/time point/age group/condition (only old-T had n=4 at 4 w.p.t.). Young (2 months) in blue and old (17 months) mice in brown; p-value: * ≤0.05 at 95% confidence interval by Mann-Whitney test. Data shown as mean ± SD. *See also Figure 2.*

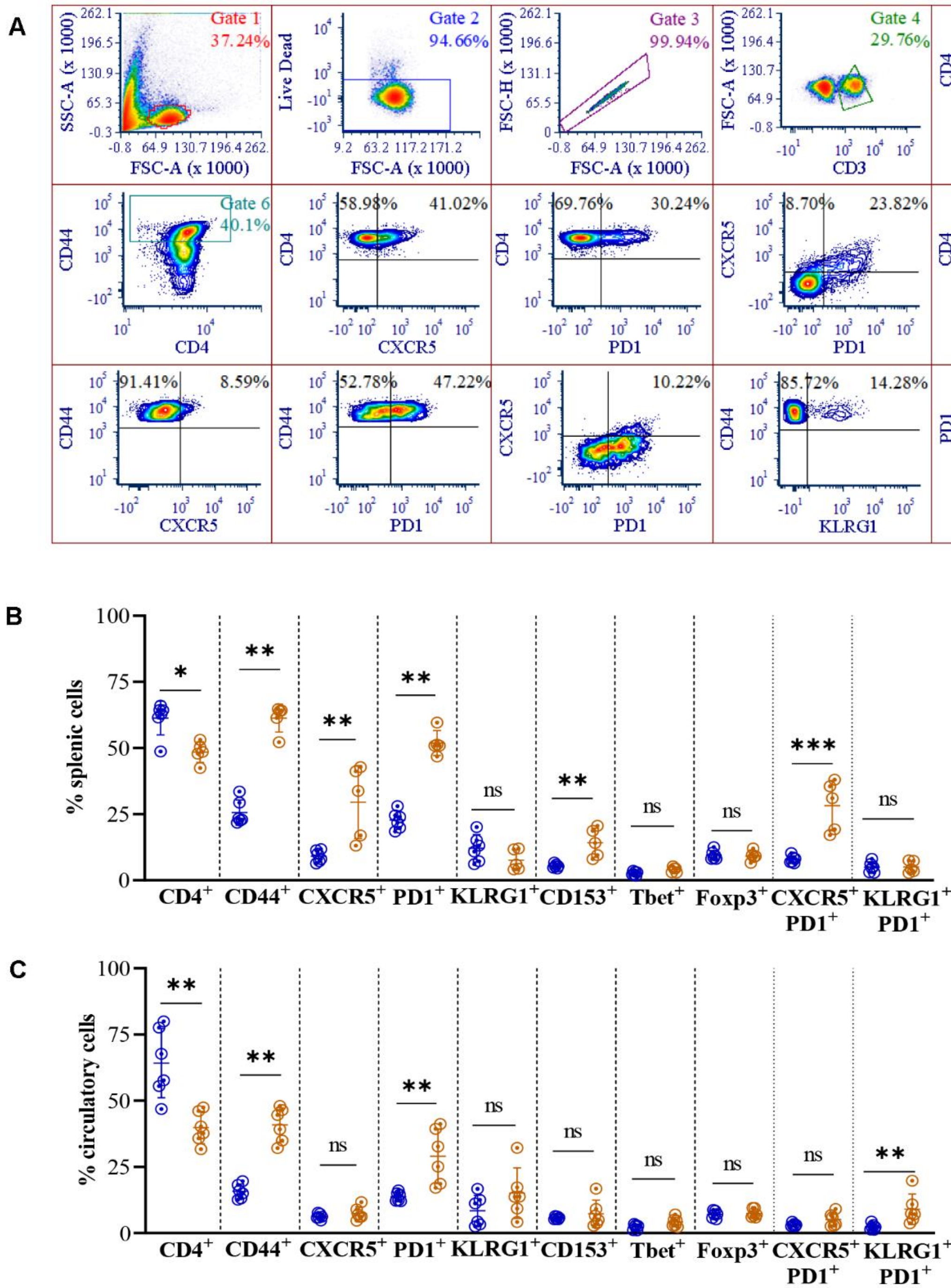

Supplementary Figure 5. Splenic and circulatory CD4+ T cell subsets frequency altered in old C57BL/6 mice. (**A**) Gating strategy in the lung of a representative mouse adopted to monitor the frequency of multiple CD4+ T cell subsets; *data analyzed using FCS Express (version 6).* Frequency of CD4+, CD4+CD44+, CD4+CXCR5+, CD4+PD1+, CD4+KLRG1+, CD4+CD153+, CD4+Tbet+, CD4+CD25+CD127-FoxP3+, CD4+CXCR5+PD1+ and CD4+KLRG1+PD1+ cells in the (**B**) spleen and (**C**) circulation of healthy mice are presented. n = 6 for young, n = 5-6 for old mice; Young (2 months) in blue and old (17 months) mice in brown; p-values: ns= non-significant, * ≤0.05, ** <0.005 and *** <0.0005 at 95% confidence interval by Mann-Whitney test. Data shown as mean ± SD.

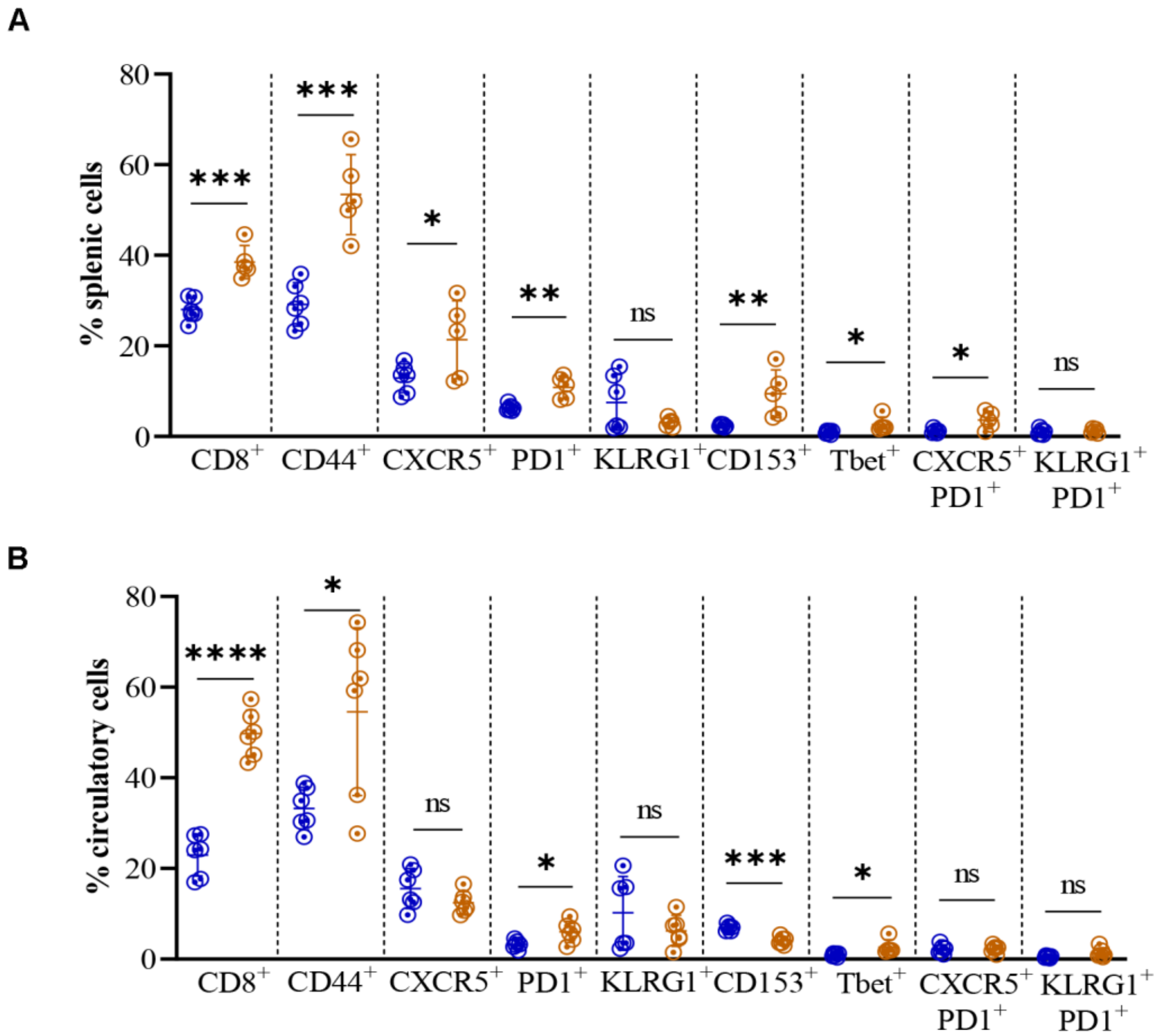

Supplementary Figure 6. Splenic and circulatory CD8+ cells subsets altered in old mice. Frequency of CD8+, CD8+CD44+, CD8+CXCR5+, CD8+PD1+, CD8+KLRG1+, CD8+CD153+, CD8+Tbet+, CD8+CXCR5+PD1+, CD8+KLRG1+PD1+cells in the (**A**) spleen and (**B**) circulation of healthy young (n=6) and old (n=5-6) mice are presented. Young (2 months) in blue and old (17 months) mice in brown; p-values: ns = non-significant, * $\leq$0.05, ** <0.005, *** <0.0005 and **** <0.0001 at 95% confidence interval by Mann- Whitney test. Data is represented as mean ± SD.

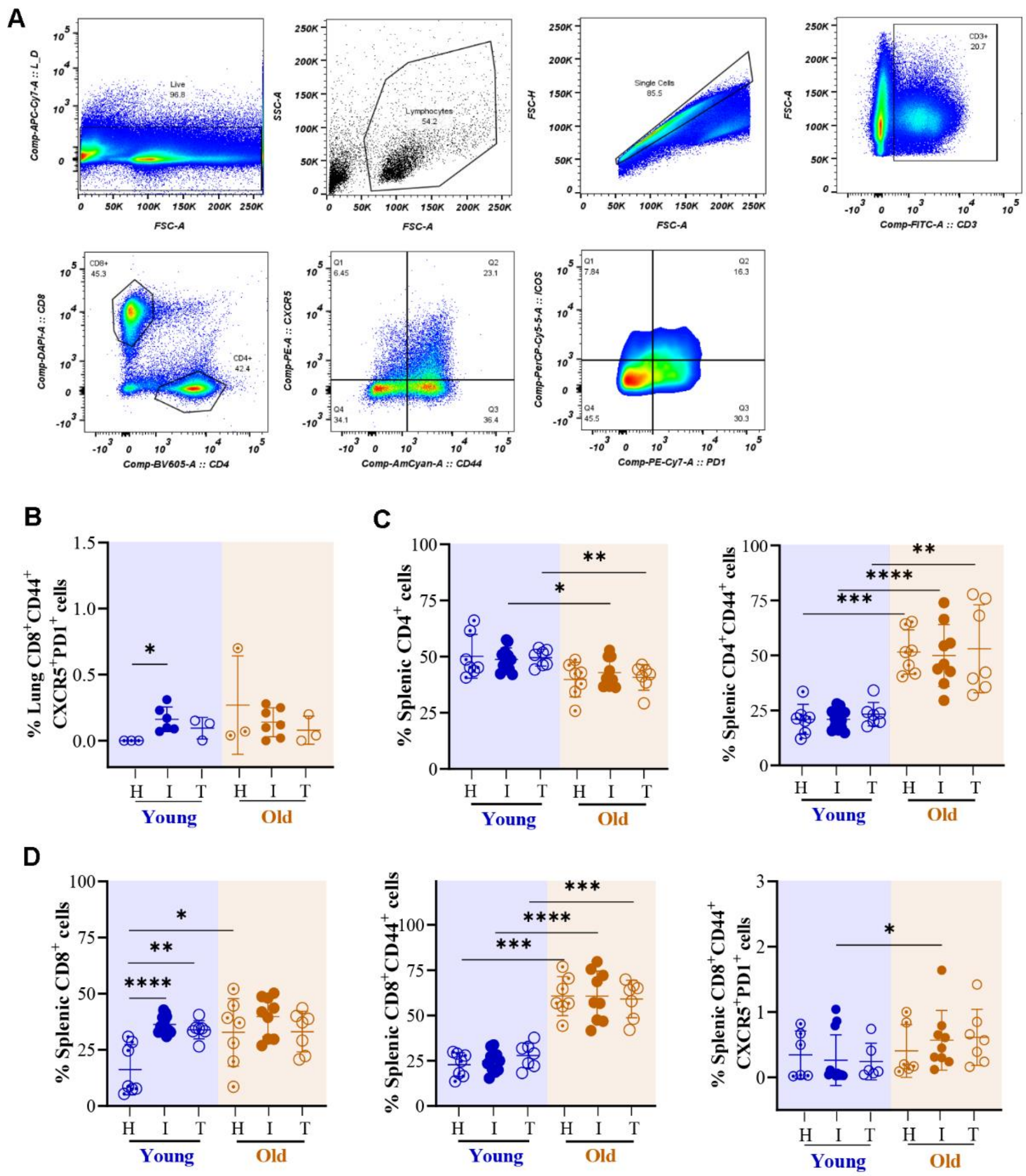

Supplementary Figure 7. Higher lung and splenic CD8+CD44+CXCR5+PD1+ cells observed in Mtb infected old mice. (**A**) Gating strategy of a representative mouse adopted to monitor the frequency of T cell subsets in the lung of C57BL/6 mice; *data analyzed using FlowJo (version 10.8).* (**B**) Frequency of lung CD8+CD44+CXCR5+PD1+ cells of healthy (H, n=3/age group), Mtb H37Rv infected (I, n= 9-10/age group) and RIF-INH treated (T, n=3/age group) C57BL/6 mice at 4 w.p.i./2 w.p.t.. Frequency of splenic (**C**) CD4+ and CD4+CD44+ (**D**) CD8+, CD8+CD44+ and CD8+CD44+CXCR5+PD1+ cells of healthy (H, n=6-7/age group), Mtb H37Rv infected (I, n=9-12/age group) and RIF-INH treated (T, n=6-7/age group) C57BL/6 mice at 4 w.p.i./2 w.p.t. Young (2-4 months) in blue and old (17-19 months) mice in brown; p-values: * ≤0.05, ** <0.005, *** <0.0005 and **** <0.0001 at 95% confidence interval by Mann-Whitney test. Data shown as mean ± SD. *See also Figure 3.*

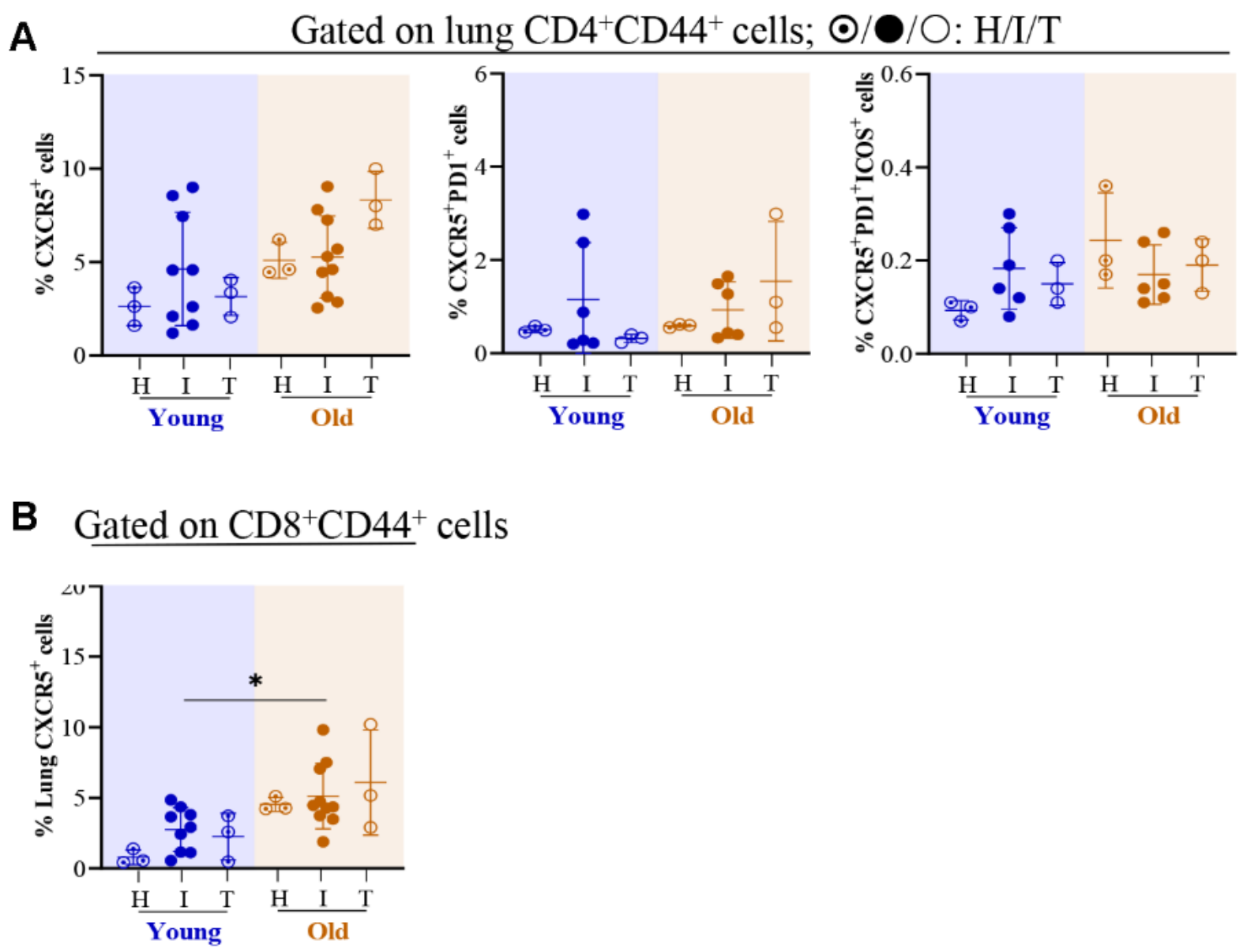

Supplementary Figure 8. Lung CD8+CD44+CXCR5+ cells were higher in old C57BL/6 mice post Mtb infection. (**A**) Frequency of CD4+CD44+CXCR5+, CD4+CD44+CXCR5+PD1+ and CD4+CD44+CXCR5+PD1+ICOS+ cells in the lungs of C57BL/6 mice at 4 w.p.i./2 w.p.t. are presented. (**B**) Frequency of CD8+CD44+CXCR5+ cells in the lungs and spleen at 4 w.p.i./2 w.p.t. are presented. Healthy (H, n=3/age group), Mtb H37Rv infected (I, n= 6-10/age group) and RIF-INH treated (T, n=3/age group). Young (2- 4 months) in blue and old (17-19 months) mice in brown; p-value: * $\leq$0.05 at 95% confidence interval by Mann-Whitney test. Data shown as mean $\pm$ SD. *See also Figure 3.*

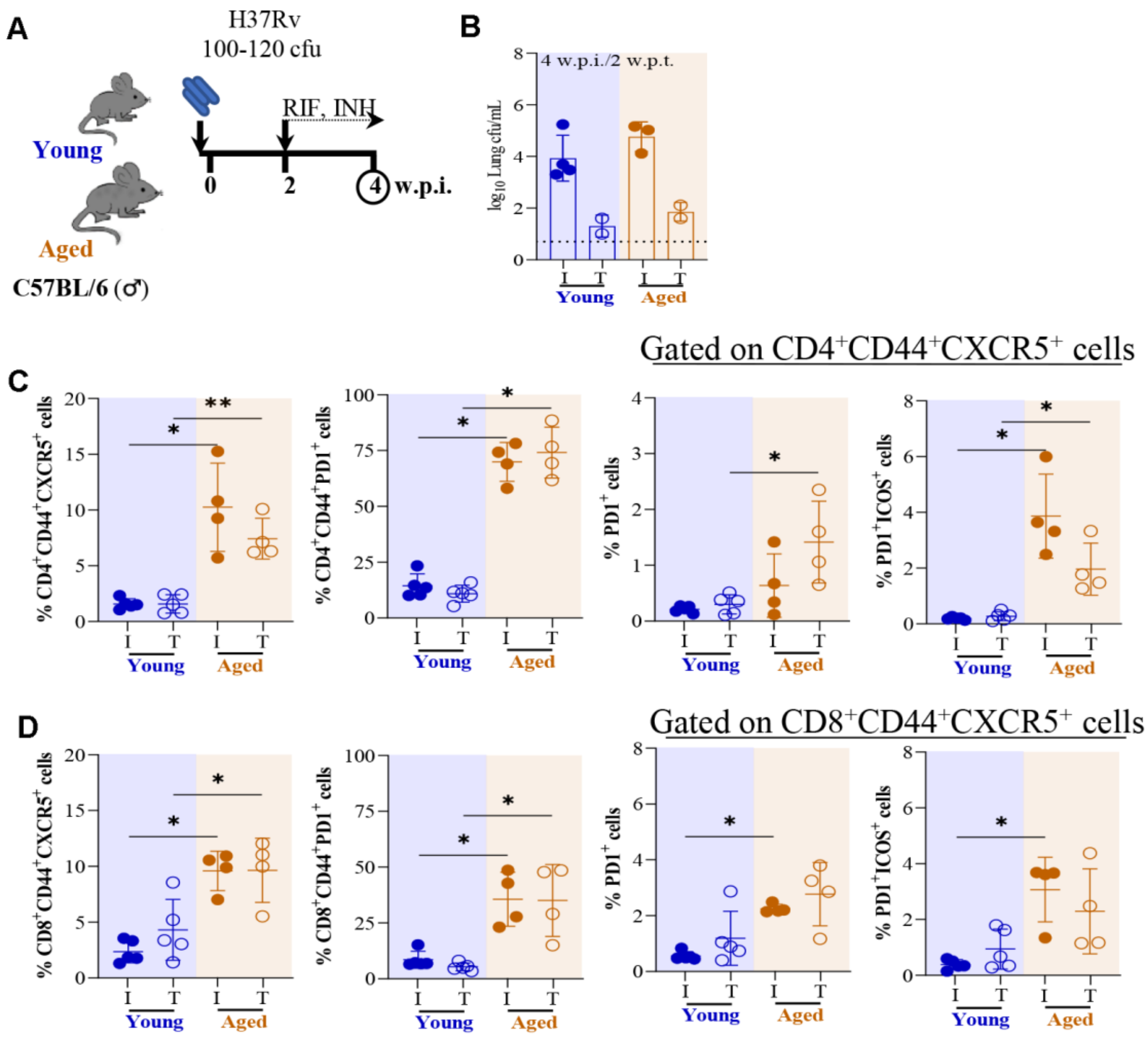

Supplementary Figure 9. Higher splenic CD4+CD44+CXCR5+ cells observed in aged mice. (**A**) Schematic of the experimental design for Mtb H37Rv infection and RIF-INH treatment of male C57BL/6 mice. (**B**) Lung bacterial burden (in log10cfu/mL) of Mtb infected mice (at 4 w.p.i.) and RIF-INH treated mice (at 2 w.p.t.; treatment started at 2 w.p.i.); dashed horizontal line represents limit of detection (LOD); Infected (I) mice: n=3-4 for young and old; Treated (T) mice: n=2 for young and old; rest cfu data points could not be collected due to plate contamination. (**C**) Frequency of splenic CD4+CD44+CXCR5+, CD4+CD44+PD1+, CD4+CD44+CXCR5+PD1+ and CD4+CD44+CXCR5+PD1+ICOS+ cells of I and T groups of mice at 4 w.p.i./2 w.p.t. (**D**) Frequency of splenic CD8+CD44+CXCR5+, CD8+CD44+PD1+, CD8+CD44+CXCR5+PD1+ and CD8+CD44+CXCR5+PD1+ICOS+ cells of I and T groups of mice at 4 w.p.i./2 w.p.t. n=4-5/condition; Young (2 months) in blue and aged (31 months) mice in brown; p-values: * ≤0.05 and ** <0.005 at 95% confidence interval by Mann-Whitney test. Data shown as mean ± SD.

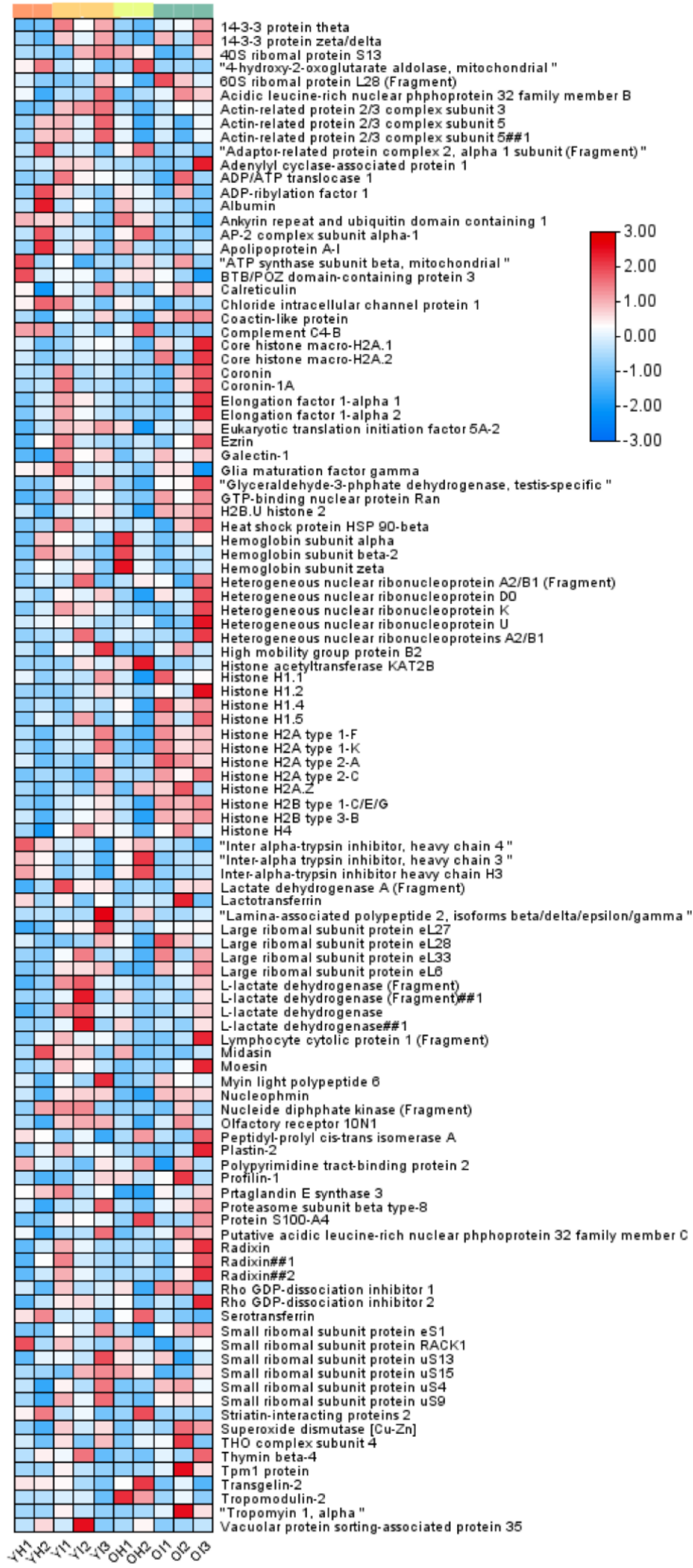

Supplementary Figure 10. Proteome distribution of CD4+CD44+ T cells changed upon Mtb infection. Heatmap showing abundance of identified splenic CD4+CD44+ T cell proteins obtained from TMT10plex experiment using healthy (H) and Mtb H37Rv infected (I) C57BL/6 mice groups (Y: young, 4 months) and (O: old, 19 months). Each row presents abundance of individual protein. 1,2,3 shows the biological replicates. Colour intensity indicates the abundance values. *See also Figure 3.*
