# Supplementary material for: Host immunosenescence compromises Mycobacterium tuberculosis clearance
Source: Aging (Albany NY). 2026 May 4;18(1):397–420. doi: 10.18632/aging.206374 (PMC13632697; doi:10.18632/aging.206374)
Supplement: Supplementary file 2 [file aging-18-1-206374-s002.pdf]

SUPPLEMENTARY TABLES

Supplementary Table 1. Compiled results of the study. Distribution of different immune cells in young (2M, 4M), old (17M, 19M) and aged (31M) male C57BL/6 mice in (A) spleen of healthy (H), Mtb H37Rv infected (I) and RIF-INH treated (T) groups at 4 w.p.i./2 w.p.t. and in (B) lung of Mtb infected mice at 4 w.p.i.; M: months. *See also Figure 3.*

A

Splenic cells	Condition	2M	4M	17M	19M	31M
CD4^{+}	H	58±9	43±2	45±4	37±8	N/A
	I	55±2	46±3	47±6	40±5	42±5
	T	49±5	49±4	43±4	41±7	43±9
CD4^{+}CD44^{+}	H	25±4	17±4	61±5	49±10	N/A
	I	23±2	20±5	65±9	42±8	88±5
	T	21±2	23±6	73±5	37±4	86±6
CD4^{+}CD44^{+}CXCR5^{+}	H	3±0.5	4±2.6	6±1.4	14±10	N/A
	I	4±0.3	4±2.3	8±1.5	16±3	10±4
	T	2±1.6	10±3	6±3.2	23±4	7.4±1.8
CD4^{+}CD44^{+}CXCR5^{+}PD1^{+}	H	3.8±1.3	1±0.7	8±4.7	3.4±2	N/A
	I	4.5±1.4	1±0.6	16±11	4.7±0.8	0.6±0.5
	T	3.8±1.2	1.3±0.1	9±3.3	5.8±1.2	1.4±0.7
CD8^{+}	H	28±2	14±13	38±4	29±16	N/A
	I	34±3	37±4	32±7	44±7	46±5
	T	29±4	35±3	24±4	40±2	42±7
CD8^{+}CD44^{+}	H	28±1	18±4	60±5	60±13	N/A
	I	29±3	23±5	56±20	62±11	77±9
	T	20±2	31±5	53±14	63±3	71±16
CD8^{+}CD44^{+} CXCR5^{+}	H	1.6±1	1.6±0.9	0.5±0.1	6±3	N/A
	I	1±0.4	2.4±1.5	4±2	8.4±3.5	9.6±1.7
	T	1.5±0.8	5±2	2±3	7.6±2.3	9.6±2.8
CD8^{+}CD44^{+}CXCR5^{+}PD1^{+}	H	0.6±0.2	0.02±0.01	1.7±1	0.2±0.3	N/A
	I	0.9±0.1	0.05±0.02	1.7±1.6	0.3±0.1	2.3±0.1
	T	0.5±0.2	0.07±0.03	0.9±0.4	0.3±0.2	2.8±1.2

B

Lung cells	2M	4M	17M	19M
CD4^{+}	28±6	41±8	26±2	48±13
CD4^{+}CD44^{+}	47±1	65±5	75±4	69±9
CD4^{+}CD44^{+}CXCR5^{+}	3±1	4±3	3.6±1.7	5±2
CD4$_{+}$CD44^{+}CXCR5^{+}PD1^{+}	6±3	1.1±1	2±1	0.8±0.6
CD8^{+}	17±3	32±9	18±4	30±7
CD8^{+}CD44^{+}	45±15	57±10	82±4	64±15
CD8^{+}CD44^{+}CXCR5^{+}	0.9±0.3	3.6±0.9	6±3	4.3±1.7
CD8^{+}CD44^{+}CXCR5^{+}PD1^{+}	4±2	0.16±0.1	2±1	0.14±0.1

Supplementary Table 2. Total number of sorted activated splenic CD4+ T cells and their protein amount from healthy young (4 months) and old (19 months) C57BL/6 mice upon Mtb H37Rv infection and RIF -INH treatment. ***See also Figure 3.***

Mice Condition	Number of $CD4^+$ $CD44^+$ cells sorted	Total protein amount (µg)
Healthy (Young)	4,31,308	24.53
	3,23,580	13.96
	4,41,483	9.06
	4,35,189	20.38
Mtb-infected (Young)	5,82,869	15.47
	3,50,937	6.79
	3,00,415	6.42
	2,80,196	16.60
	1,63,427	7.17
	2,00,368	20.00
RIF-INH treated (Young)	2,47,515	13.58
	3,20,773	24.91
	6,49,437	18.11
	1,36,499	1.13
	5,18,395	6.79
	5,34,188	10.94
	3,61,425	15.47
Healthy (Old)		
	10,17,677	19.25
	6,63,651	13.21
Mtb-infected (Old)	4,55,972	8.30
	4,37,661	14.72
	4,28,506	12.45
	6,22,087	20.38
	6,84,220	10.94
	3,67,149	10.57
RIF-INH treated (Old)	7,38,297	6.79
	3,66,620	36.23
	1,99,573	36.60
	3,51,695	8.68
