# Supplementary material for: Host immunosenescence compromises Mycobacterium tuberculosis clearance
Source: Aging (Albany NY). 2026 May 4;18(1):397–420. doi: 10.18632/aging.206374 (PMC13632697; doi:10.18632/aging.206374)
Supplement: Supplementary file 3 [file aging-18-1-206374-s003.pdf]

Supplementary Table 3. List of identified proteins (n= 109) from splenic $CD4^+CD44^+$ T cells (TMT- Set 1) showing Mtb H37Rv infected young and old C57BL/6 mice with their respective healthy controls. Y: young (4 months); O: old (19 months); I: Mtb infected; H: Healthy. *See also Figure 3.*

Accession	Identified Protein(s) name	log_2fold change							
		**YI1/YH2**	**YI2/YH2**	**YI3/YH2**	**YH1/YH2**	**OI1/OH2**	**OI2/OH2**	**OI3/OH2**	**OH1/OH2**
P68254	14-3-3 protein theta	1.66	1.01	1.46	-0.12	0.95	1.12	1.66	0.47
P63101	14-3-3 protein zeta/delta	2.12	1.53	2.35	0.86	2.32	1.17	2.58	0.70
Q921R2	40S ribosomal protein S13	-0.27	1.00	1.19	0.11	-1.35	-1.03	0.08	0.33
Q9DCU9	4-hydroxy-2-oxoglutarate aldolase, mitochondrial	-1.45	-0.98	-2.35	-0.75	-2.04	-1.97	-2.13	-2.09
F6Z0X0	60S ribosomal protein L28 (Fragment)	-0.16	0.31	1.75	0.92	3.42	2.73	2.05	2.23
Q9EST5	Acidic leucine-rich nuclear phosphoprotein 32 family member B	0.94	0.97	2.05	1.31	0.32	0.99	0.70	-0.52
Q9JM76	Actin-related protein 2/3 complex subunit 3	1.86	2.12	2.31	-0.11	1.30	1.85	1.73	1.26
Q3UA72	Actin-related protein 2/3 complex subunit 5	0.04	-0.36	0.33	-0.70	0.78	0.13	0.92	0.86
Q9CPW4	Actin-related protein 2/3 complex subunit 5	0.04	-0.36	0.33	-0.70	0.78	0.13	0.92	0.86
A0A140LIG7	Adaptor-related protein complex 2, alpha 1 subunit (Fragment)	-1.96	-1.71	-3.40	-2.08	-2.80	-2.06	-3.14	-1.02
P40124	Adenylyl cyclase-associated protein 1	0.79	0.74	-0.15	-0.31	-0.49	-0.20	2.45	0.21
P48962	ADP/ATP translocase 1	1.60	0.89	0.77	-0.23	-1.22	1.49	-0.22	0.49
P84078	ADP-ribosylation factor 1	-0.72	-1.43	-2.10	-1.86	-1.19	0.60	-1.15	0.32
P07724	Albumin	-3.24	-1.89	-4.74	-3.08	-0.57	-2.00	-2.07	1.12
A0A5F8MPK3	Ankyrin repeat and ubiquitin domain containing 1	-0.03	-1.30	-2.06	0.23	-1.53	-1.16	-3.56	0.66
P17426	AP-2 complex subunit alpha-1	-1.96	-1.71	-3.40	-2.08	-2.80	-2.06	-3.14	-1.02
Q00623	Apolipoprotein A-I	-2.97	-1.41	-6.18	-4.63	-1.61	-1.28	1.08	2.50
P56480	ATP synthase subunit beta, mitochondrial	0.30	-0.30	0.04	0.76	-0.30	0.14	-0.46	-0.43
P58545	BTB/POZ domain-containing protein 3	0.25	0.28	-1.00	1.42	-0.28	-1.08	-3.33	-0.05
P14211	Calreticulin	2.17	1.96	2.82	2.33	1.17	1.58	1.26	0.47
Q9Z1Q5	Chloride intracellular channel protein 1	-0.15	-1.05	-1.87	-0.71	-1.20	-0.36	-0.60	0.35
Q9CQI6	Coactosin-like protein	2.00	1.50	2.43	1.31	2.37	2.82	2.83	1.13

P01029	Complement C4-B	-1.79	-1.02	-2.01	-0.05	-2.34	-1.99	-2.26	-1.14
Q9QZQ8	Core histone macro-H2A.1	0.32	0.74	0.85	0.68	1.16	0.71	1.81	0.77
Q8CCK0	Core histone macro-H2A.2	0.51	0.96	0.90	0.86	1.85	-0.16	2.20	0.49
G3UYK8	Coronin	1.95	-0.02	0.52	0.14	-1.26	1.92	2.62	-0.28
O89053	Coronin-1A	1.68	-0.16	-0.14	-0.36	-1.24	1.23	2.12	-0.37
P10126	Elongation factor 1-alpha 1	1.24	0.67	-0.03	-1.32	-0.51	0.79	2.72	0.07
P62631	Elongation factor 1-alpha 2	1.12	0.29	-0.20	-1.41	-0.79	0.77	2.51	-0.34
Q8BGY2	Eukaryotic translation initiation factor 5A-2	0.83	0.71	1.02	-0.91	2.04	1.91	2.50	2.52
P26040	Ezrin	0.75	-0.39	-0.67	-1.72	-0.81	0.42	1.38	-0.50
P16045	Galectin-1	3.21	2.53	2.83	0.62	0.85	0.27	0.73	-1.11
Q9ERL7	Glia maturation factor gamma	0.62	-0.53	-0.37	-0.06	1.11	1.09	-1.25	0.52
Q64467	Glyceraldehyde-3-phosphate dehydrogenase, testis-specific	1.52	1.20	1.90	-0.07	2.64	2.73	3.81	1.56
P62827	GTP-binding nuclear protein Ran	1.70	0.75	1.05	-0.49	1.66	1.08	1.76	0.31
Q9D2U9	H2B.U histone 2	1.43	1.72	2.04	1.38	3.09	2.96	3.25	2.02
P11499	Heat shock protein HSP 90-beta	1.52	0.31	0.59	-0.43	-1.68	0.64	1.22	-0.44
P01942	Hemoglobin subunit alpha	-0.61	-0.84	-3.25	-3.36	-2.11	-0.27	0.80	2.36
P02089	Hemoglobin subunit beta-2	-0.41	-1.61	-2.36	-2.36	-1.53	0.04	-0.26	1.65
P06467	Hemoglobin subunit zeta	-0.51	0.28	-3.73	-2.75	-3.19	-2.34	-0.59	2.53
A0A0N4SUM2	Heterogeneous nuclear ribonucleoprotein A2/B1 (Fragment)	-0.44	1.10	-1.04	-0.84	-0.17	-1.87	0.71	-0.66
Q60668	Heterogeneous nuclear ribonucleoprotein D0	1.18	0.84	1.46	0.71	3.20	2.60	4.06	2.05
H3BLL4	Heterogeneous nuclear ribonucleoprotein K	0.91	0.61	0.12	-0.78	-0.09	0.76	2.32	0.58
Q8VEK3	Heterogeneous nuclear ribonucleoprotein U	0.30	-0.12	-0.80	-0.27	-2.58	-1.64	1.66	-0.61
O88569	Heterogeneous nuclear ribonucleoproteins A2/B1	0.41	3.24	-0.34	-1.07	-0.38	-1.31	3.30	0.82
P30681	High mobility group protein B2	2.17	1.67	3.63	1.34	2.29	3.44	1.87	0.16
Q9JHD1	Histone acetyltransferase KAT2B	-0.82	5.28	3.75	-0.37	-9.30	-7.78	-4.06	-1.77
P43275	Histone H1.1	0.68	0.54	1.83	0.96	4.63	3.44	3.65	3.18
P15864	Histone H1.2	-0.10	1.78	3.26	0.38	2.67	1.43	4.77	1.72
P43274	Histone H1.4	-0.76	0.30	-0.19	-0.45	4.92	4.04	4.52	3.79
P43276	Histone H1.5	-0.97	1.11	-1.31	-1.50	4.62	3.14	5.11	3.57

Q8CGP5	Histone H2A type 1-F	1.08	1.13	2.34	0.84	2.67	2.19	2.39	0.81
Q8CGP7	Histone H2A type 1-K	1.08	1.13	2.34	0.84	2.67	2.19	2.39	0.81
Q6GSS7	Histone H2A type 2-A	1.35	0.20	2.91	2.16	2.38	2.02	1.48	-0.74
Q64523	Histone H2A type 2-C	-0.03	-0.35	0.81	-0.25	0.83	0.48	0.97	0.11
P0C0S6	Histone H2A.Z	0.85	0.33	1.64	0.58	-0.11	0.51	-1.00	-1.67
Q6ZWY9	Histone H2B type 1-C/E/G	1.08	1.40	1.69	0.51	2.66	2.54	2.83	1.44
Q8CGP0	Histone H2B type 3-B	1.43	1.72	2.04	1.38	3.09	2.96	3.25	2.02
P62806	Histone H4	2.17	2.68	2.25	1.50	1.25	1.82	1.05	1.11
A6X935	Inter alpha-trypsin inhibitor, heavy chain 4	-0.70	-0.54	-2.38	0.63	-1.01	-1.31	-2.37	-0.34
A0A2I3BRQ3	Inter-alpha trypsin inhibitor, heavy chain 3	-0.83	-0.24	-1.44	0.30	-1.83	-1.40	-1.25	-1.00
Q61704	Inter-alpha-trypsin inhibitor heavy chain H3	-0.94	-0.39	-1.62	0.38	-1.68	-1.48	-1.32	-0.75
A0A1B0GSL7	Lactate dehydrogenase A (Fragment)	1.62	0.78	0.83	-1.14	-0.25	0.92	0.94	-0.23
P08071	Lactotransferrin	1.80	-1.20	1.62	2.06	-0.36	2.40	-2.68	-1.57
Q61029	Lamina-associated polypeptide 2, isoforms beta/delta/epsilon/gamma	-1.26	-1.06	5.25	-0.54	-4.81	-4.59	-1.97	-3.82
P61358	Large ribosomal subunit protein eL27	2.33	2.35	3.57	-1.40	1.21	1.27	1.45	0.91
P41105	Large ribosomal subunit protein eL28	-0.16	0.31	1.75	0.92	3.42	2.73	2.05	2.23
O55142	Large ribosomal subunit protein eL33	1.00	1.83	0.37	0.18	2.55	1.03	2.34	1.45
P47911	Large ribosomal subunit protein eL6	0.75	0.78	0.92	-0.14	1.45	1.06	1.67	0.13
D3YZQ9	L-lactate dehydrogenase (Fragment)	1.14	1.35	0.46	-0.49	0.13	0.49	0.96	0.50
A0A0N4SVV8	L-lactate dehydrogenase (Fragment)	0.58	1.80	0.06	0.00	0.46	0.01	0.97	1.04
A0A1B0GQX5	L-lactate dehydrogenase	1.14	1.35	0.46	-0.49	0.13	0.49	0.96	0.50
A0A6I8MX27	L-lactate dehydrogenase	0.58	1.80	0.06	0.00	0.46	0.01	0.97	1.04
D3Z311	Lymphocyte cytosolic protein 1 (Fragment)	1.00	-0.04	0.11	-2.06	-0.42	1.47	4.36	1.08
A2ANY6	Midasin	-0.92	-0.68	-2.32	-1.98	0.58	0.06	1.12	2.28
P26041	Moesin	0.92	0.54	0.01	-0.27	-0.18	0.63	1.74	-0.38
Q60605	Myosin light polypeptide 6	0.98	0.48	1.86	0.79	0.81	0.56	0.44	-0.26
Q61937	Nucleophosmin	1.98	2.10	2.10	1.34	2.93	2.89	2.76	1.06
Q5NC80	Nucleoside diphosphate kinase (Fragment)	0.09	0.11	-1.03	-1.05	-0.34	0.80	-0.04	-0.11
Q60887	Olfactory receptor 10N1	2.66	2.87	2.82	1.51	1.97	3.03	1.89	1.74
P17742	Peptidyl-prolyl cis-trans isomerase A	-0.75	-0.13	-1.50	0.16	-0.93	-1.23	0.25	-1.02

Q61233	Plastin-2	1.00	-0.04	0.11	-2.06	-0.42	1.47	4.36	1.08
Q91Z31	Polypyrimidine tract-binding protein 2	-0.13	-0.38	0.17	0.32	-1.12	-0.08	-0.56	-0.42
P62962	Profilin-1	0.82	2.07	2.77	1.64	0.68	1.90	-0.10	0.94
D3Z7C6	Prostaglandin E synthase 3	0.22	-0.50	-0.23	-0.20	1.18	0.93	1.34	0.10
P28063	Proteasome subunit beta type-8	2.01	2.25	3.74	2.41	1.33	1.75	2.28	0.86
P07091	Protein S100-A4	2.46	2.31	2.02	-0.74	-3.14	-2.93	-0.49	-3.41
Q64G17	Putative acidic leucine-rich nuclear phosphoprotein 32 family member C	0.94	0.97	2.05	1.31	0.32	0.99	0.70	-0.52
A0A5F8MPB9	Radixin	0.89	0.23	-0.31	-1.14	-0.33	0.85	1.81	0.04
Q7TSG6	Radixin	0.75	-0.39	-0.67	-1.72	-0.81	0.42	1.38	-0.50
P26043	Radixin	0.89	0.23	-0.31	-1.14	-0.33	0.85	1.81	0.04
Q99PT1	Rho GDP-dissociation inhibitor 1	1.38	0.67	0.86	0.84	2.01	1.96	0.82	1.60
Q61599	Rho GDP-dissociation inhibitor 2	0.60	0.73	0.16	-1.15	0.80	0.36	2.36	1.20
Q921I1	Serotransferrin	-1.73	-1.43	-3.51	-0.72	-2.23	-3.38	-4.54	-1.28
P97351	Small ribosomal subunit protein eS1	0.79	0.54	1.23	0.01	1.30	1.63	1.78	0.90
P68040	Small ribosomal subunit protein RACK1	0.93	0.20	-0.20	1.49	-1.44	-0.42	0.31	0.79
P62270	Small ribosomal subunit protein uS13	0.06	-0.16	0.79	-0.74	0.39	-1.01	-0.07	0.26
P62301	Small ribosomal subunit protein uS15	-0.27	1.00	1.19	0.11	-1.35	-1.03	0.08	0.33
Q6ZWN5	Small ribosomal subunit protein uS4	2.33	1.65	3.03	1.76	1.31	1.47	0.79	-0.29
P14131	Small ribosomal subunit protein uS9	2.26	1.66	2.58	1.08	1.32	1.46	1.31	0.34
Q8C9H6	Striatin-interacting proteins 2	-1.45	-0.98	-2.35	-0.75	-2.04	-1.97	-2.13	-2.09
P08228	Superoxide dismutase [Cu-Zn]	2.86	2.05	2.74	1.35	-0.53	1.48	1.20	-0.97
O08583	THO complex subunit 4	2.41	0.45	2.70	1.66	0.12	1.60	-1.54	-1.48
P20065	Thymosin beta-4	-0.27	0.88	-2.37	-0.93	1.57	0.74	2.95	0.62
B7ZNL3	Tpm1 protein	0.93	0.32	-0.12	0.06	0.54	2.03	0.95	-0.23
Q9WVA4	Transgelin-2	-0.07	-0.61	-0.96	0.03	-1.77	-1.04	-2.12	-0.89
Q9JKK7	Tropomodulin-2	0.65	0.63	-2.90	-0.08	-2.78	-1.25	-3.31	0.80
Q8BSH3	Tropomyosin 1, alpha	0.93	0.32	-0.12	0.06	0.54	2.03	0.95	-0.23
Q9EQH3	Vacuolar protein sorting-associated protein 35	-1.09	1.47	-2.61	-1.38	-2.28	-1.66	-0.99	-1.45
