# Supplementary material for: Host immunosenescence compromises Mycobacterium tuberculosis clearance
Source: Aging (Albany NY). 2026 May 4;18(1):397–420. doi: 10.18632/aging.206374 (PMC13632697; doi:10.18632/aging.206374)
Supplement: Supplementary file 4 [file aging-18-1-206374-s004.pdf]

Supplementary Table 4. List of identified proteins (n=41) from splenic $CD4^{+}CD44^{+}$ T cells (TMT- Set 2) showing RIF-INH treated young and old C57BL/6 mice with Mtb H37Rv infected controls. Y: young (4 months); O: old (19 months); T: RIF-INH treated; I: Mtb infected. *See also Figure 3.*

Accession	Identified Protein(s) name	log_2fold change							
		**YT1/YI1**	**YT2/YI1**	**YT3/YI1**	**YI2/YI1**	**OT1/OI1**	**OT2/OI1**	**OT3/OI1**	**OI2/OI1**
P50247	Adenosylhomocysteinase	-1.05	0.04	-4.26	-1.06	-1.85	0.55	1.47	-0.50
P32020	Sterol carrier protein 2	-0.88	-1.28	-3.18	-0.32	-1.27	-0.80	0.37	-1.20
A0A0G2JGN4	Small nuclear ribonucleoprotein B	-1.17	-1.59	-2.98	-0.86	-1.27	-1.28	1.24	0.24
S4R1W1	Glyceraldehyde-3-phosphate dehydrogenase	-2.30	-2.27	-1.51	-1.88	-0.41	-0.22	-1.00	0.50
O08583	THO complex subunit 4	-0.53	-0.80	-0.01	0.54	-3.02	-3.39	-0.11	-0.55
P62806	Histone H4	-1.29	-3.34	-3.27	-0.02	-1.88	-3.44	-0.43	-1.12
Q64524	Histone H2B type 2-E	-0.70	-1.60	-2.52	0.07	-2.39	-4.07	0.24	0.10
F8WI35	Histone H3	-2.11	-2.57	-2.79	-0.18	-1.91	-3.82	-1.45	0.48
E0CZ27	Histone H3 (Fragment)	-2.18	-2.62	-2.80	-0.19	-1.86	-3.80	-1.44	0.53
Q64525	Histone H2B type 2-B	-1.24	-2.24	-2.69	-0.16	-1.75	-3.49	-1.25	0.62
Q8CGP2	Histone H2B type 1-P	-0.96	-1.63	-2.41	0.05	-2.00	-3.58	-0.13	0.24
Q9D2U9	H2B.U histone 2	-0.94	-1.79	-2.60	0.01	-2.03	-3.88	0.00	0.20
E0CYR7	H3.3 histone A (Fragment)	-2.11	-2.65	-2.87	-0.20	-1.71	-3.76	-1.34	0.57
Q8CGP0	Histone H2B type 3-B	-0.94	-1.79	-2.60	0.01	-2.03	-3.88	0.00	0.20
P70696	Histone H2B type 1-A	-0.96	-1.65	-2.36	0.04	-1.95	-3.53	-0.17	0.25
A0A8I4SYN6	Histone H3	-2.00	-2.43	-2.66	-0.15	-1.51	-3.78	-1.50	0.36
P27661	Histone H2AX	-2.19	-2.60	-2.65	-0.37	-1.17	-3.93	-0.60	0.91
P07309	Transthyretin	0.39	0.03	0.55	-0.69	1.41	1.74	0.88	-0.61
V9GX81	Maestro heat-like repeat family member 6	-0.35	-0.12	-0.64	0.21	0.04	-0.17	-1.56	-0.12
P15864	Histone H1.2	-0.99	-0.48	-1.84	0.27	-2.37	-4.25	-3.62	0.06
Q07133	Histone H1t	-1.41	-0.61	-2.62	0.14	-2.12	-5.30	-4.39	0.09
P43275	Histone H1.1	-0.49	-0.29	-1.86	0.56	-1.61	-3.81	-4.33	0.12
G3UWL7	Histone H2A	-2.03	-2.53	-2.37	-0.67	0.52	-4.01	-0.79	1.19

B2RY04	Dedicator of cytokinesis protein 5	-0.06	-0.51	0.80	0.31	2.06	2.02		-0.65
A8DUK4	Beta-globin	6.13	5.47	3.53	-0.15	-1.76	1.11	2.33	-1.13
A0A0U1RQ96	Actin, gamma 2, smooth muscle, enteric (Fragment)	-4.50	-4.81	-4.38	-2.37	-4.75	-3.76	-3.22	0.85
Q8BFZ3	Beta-actin-like protein 2	-3.66	-3.91	-4.29	-2.11	-3.98	-3.68	-2.62	0.79
E9Q5F4	Actin, beta (Fragment)	-3.91	-3.75	-1.03	-1.72	-2.24	-3.14	-2.50	0.38
E9Q1F2	Actin, beta	-4.02	-3.82	-0.94	-1.72	-2.21	-3.12	-2.55	0.36
P63268	Actin, gamma-enteric smooth muscle	-3.67	-3.60	-0.67	-1.64	-2.18	-3.22	-2.43	0.21
A0A1D5RM20	Actin alpha 1, skeletal muscle (Fragment)	-3.56	-3.46	-0.41	-1.55	-1.96	-3.15	-2.33	0.10
F6WX90	Actin, alpha, cardiac muscle 1 (Fragment)	-3.67	-3.53	-0.28	-1.55	-1.90	-3.13	-2.39	0.06
A0A338P692	Alpha-2-HS-glycoprotein (Fragment)	-0.79	-0.31	-0.47	0.10	0.78	1.85	-3.15	-0.10
Q6NS59	Protein FAM135A	0.34	0.44	0.37	0.42	0.90	1.98	-4.33	-0.17
Q3T052	Inter-alpha-trypsin inhibitor heavy chain H4	-0.57	-0.34	-0.06	0.09	1.17	0.37	-3.07	0.25
P06467	Hemoglobin subunit zeta	-0.57	-2.06	-3.20	-0.70	0.75	3.25	-0.33	-0.41
F7CJN9	Transferrin (Fragment)	-0.28	0.59	0.74	0.09	2.19	2.57	-2.00	-0.21
Q921I1	Serotransferrin	0.40	0.87	0.52	0.09	1.96	1.85	-0.36	-0.45
P57016	Ladinin-1	0.74	0.77	0.81	-0.54	2.30	1.96	1.31	-1.21
P05202	Aspartate aminotransferase, mitochondrial	-0.63	0.50	1.24	0.58	3.23	2.87	3.35	-0.24
D3YYR8	Transferrin (Fragment)	2.24	1.89	-0.89	0.07	1.50	-0.47	1.17	-0.97
